# HIF1A Reduces Acute Lung Injury by Optimizing Carbohydrate Metabolism in the Alveolar Epithelium

**DOI:** 10.1371/journal.pbio.1001665

**Published:** 2013-09-24

**Authors:** Tobias Eckle, Kelley Brodsky, Megan Bonney, Thomas Packard, Jun Han, Christoph H. Borchers, Thomas J. Mariani, Douglas J. Kominsky, Michel Mittelbronn, Holger K. Eltzschig

**Affiliations:** 1 Organ Protection Program, Department of Anesthesiology, University of Colorado School of Medicine, Denver, Colorado, United States of America; 2 University of Victoria–Genome BC Proteomics Centre, Department of Biochemistry and Microbiology, University of Victoria, Victoria, British Columbia, Canada; 3 University of Rochester, Center for Pediatric Biomedical Research, Rochester, New York, United States of America; 4 Institute of Neurology (Edinger Institute), University of Frankfurt, Frankfurt, Germany; University of Cambridge, United Kingdom

## Abstract

**Background:**

While acute lung injury (ALI) contributes significantly to critical illness, it resolves spontaneously in many instances. The majority of patients experiencing ALI require mechanical ventilation. Therefore, we hypothesized that mechanical ventilation and concomitant stretch-exposure of pulmonary epithelia could activate endogenous pathways important in lung protection.

**Methods and Findings:**

To examine transcriptional responses during ALI, we exposed pulmonary epithelia to cyclic mechanical stretch conditions—an in vitro model resembling mechanical ventilation. A genome-wide screen revealed a transcriptional response similar to hypoxia signaling. Surprisingly, we found that stabilization of hypoxia-inducible factor 1A (HIF1A) during stretch conditions in vitro or during ventilator-induced ALI in vivo occurs under normoxic conditions. Extension of these findings identified a functional role for stretch-induced inhibition of succinate dehydrogenase (SDH) in mediating normoxic HIF1A stabilization, concomitant increases in glycolytic capacity, and improved tricarboxylic acid (TCA) cycle function. Pharmacologic studies with HIF activator or inhibitor treatment implicated HIF1A-stabilization in attenuating pulmonary edema and lung inflammation during ALI in vivo. Systematic deletion of HIF1A in the lungs, endothelia, myeloid cells, or pulmonary epithelia linked these findings to alveolar-epithelial HIF1A. In vivo analysis of ^13^C-glucose metabolites utilizing liquid-chromatography tandem mass-spectrometry demonstrated that increases in glycolytic capacity, improvement of mitochondrial respiration, and concomitant attenuation of lung inflammation during ALI were specific for alveolar-epithelial expressed HIF1A.

**Conclusions:**

These studies reveal a surprising role for HIF1A in lung protection during ALI, where normoxic HIF1A stabilization and HIF-dependent control of alveolar-epithelial glucose metabolism function as an endogenous feedback loop to dampen lung inflammation.

## Abstract 2

A study of acute lung injury reveals the involvement of transcription factor HIF1A in lung protection, where normoxic HIF1A stabilization functions to control alveolar epithelial glucose metabolism.

## Author Summary

Acute lung injury is a devastating lung disease caused by injuries or acute infections to the lung. In patients it manifests itself as acute respiratory distress syndrome. Severe pulmonary edema and uncontrolled lung inflammation are typical symptoms of acute lung injury, which make it hard for patients to breath efficiently. In the clinical course of the disease, patients require mechanical ventilation to support their lung function and to provide sufficient oxygen levels to vital organs such as the brain, the heart, or the kidneys. We hypothesized that stretch conditions—such as those that occur during mechanical ventilation—result in transcriptional adaptation of alveolar epithelial cells—the innermost lining of the lungs. In this study we identified an unexpected involvement of the transcription factor hypoxia-inducible factor HIF1A in lung protection. We observed that during acute lung injury, stabilization of HIF1A is mediated by increased levels of succinate, an intermediate product of the citrate cycle. Interestingly, we show that HIF1A in alveolar epithelia functions to induce a transcriptional program that improves the efficiency of carbohydrate metabolism by injured lungs, thereby helping to adapt to the injurious conditions of mechanical stretch and to reduce lung inflammation. These preclinical findings highlight the potential for pharmacological HIF1A stabilization in the treatment of acute lung injury.

## Introduction

Acute lung injury (ALI) is an inflammatory disease of the lungs that is characterized by hypoxemic respiratory failure with bilateral pulmonary infiltrates, not attributable to left heart failure [1]–[4]. Among the hallmarks of ALI are severe arterial hypoxemia and uncontrolled accumulation of inflammatory cells into different compartments of the lungs, in conjunction with cytokine release and inflammatory activation of recruited or resident cells. Particularly alveolar epithelial injury plays a key role in the pathogenesis of ALI, leading to disruptions of the alveolar-capillary barrier function, resulting in extensive pulmonary edema, attenuated gas exchange, and uncontrolled lung inflammation [2]. While there is currently no specific therapy available, management consists of aggressive treatment of the initiating cause, vigilant supportive care, and the prevention of nosocomial infections. From a clinical perspective, it is important to point out that ALI is among the leading causes of morbidity and mortality of patients requiring critical care medicine. For example, a large scale prospective, population-based, cohort study indicates that each year in the United States there are close to 200,000 cases of ALI, which are associated with 74,500 deaths and 3.6 million hospital days [5]. Moreover, long-term disabilities in ALI survivors are significant. A landmark study who followed ALI survivors over 5 years revealed that exercise limitation, physical and psychological sequelae, decreased physical quality of life, as well as increased costs and use of health care services are important legacies of severe lung injury [6]. Taken together, these studies highlight the importance for finding novel ALI treatment approaches with the goal to dampen excessive lung inflammation [2],[7]–[9] or to support its resolution phase [10]–[16].

While ALI has a major impact on the morbidity and mortality of patients requiring critical care medicine and mechanically ventilation, many episodes of ALI are self-limiting, and resolve spontaneously through molecular pathways that are transcriptionally controlled [10]–[12],[17]. In spite of profound inflammatory responses to surgery and mechanical ventilation [18], patients undergoing major thoracic surgery for lung cancer have an overall incidence of ALI of less than 5% [19], open heart surgery with cardiopulmonary bypass less than 0.5% [20], or kidney transplantation of less than 0.2% [21]. Based on these clinical observations, we hypothesized that innate adaptive pathways exist that dampen acute pulmonary edema and lung inflammation during ALI in mechanically ventilated patients.

To identify novel endogenous pathways important for lung protection and ALI resolution that are turned on by mechanical ventilation, we utilized stretch exposure of pulmonary epithelial cells as a single-cell-based in vitro model for ventilator-induced ALI [22],[23]. Very surprisingly, genome-wide microarray screening for alterations in gene transcription of human pulmonary epithelial cells exposed to cyclic mechanical stretch conditions revealed a transcriptional response that shared many similarities with ambient hypoxia exposure. Subsequent studies identified a molecular pathway resulting in normoxic stabilization of the transcription factor hypoxia-inducible factor HIF1A during stretch conditions in vitro or during ALI induced by mechanical ventilation in vivo.

HIF is a transcription factor that is critical for tissue adaptation to conditions of hypoxia [4],[24]–[31]. More recently, HIF has also been implicated in the control of immune responses during inflammatory processes including ischemia and reperfusion, cancer or mucosal inflammation [3],[4],[32]–[34]. Sites of acute inflammation are frequently characterized by considerable shifts in the supply and demand of metabolites that result in limited oxygen availability (inflammation-associated hypoxia) [7],[35]–[38]. Consistent with a role for hypoxia-signaling in cellular adaptation to conditions of limited oxygen availability [31],[36],[39], several studies implicate hypoxia-dependent signaling pathways in the attenuation of mucosal inflammation [7],[33],[40]–[43]. Surprisingly and in contrast to some of the above studies on inflammatory hypoxia [36],[37],[44],[45], we observed that pulmonary stabilization of HIF1A during ALI occurs under normoxic conditions. Consistent with previous studies [46]–[48], our findings implicate a metabolic pathway in normoxic HIF stabilization, including stretch-induced inhibition of succinate dehydrogenase (SDH) activity. In fact, previous studies had indicated that normoxic stabilization of HIF in cancer involves inhibition or mutation of the metabolic enzyme SDH [46],[48]. Extension of these initial findings utilizing pharmacologic and genetic models of hypoxia-signaling during ALI revealed that HIF controls a functional link between alveolar epithelial metabolism and lung inflammation during ALI, thus implicating pharmacologic strategies to activate these HIF-dependent metabolic pathways for ALI treatment. Indeed, compounds such as HIF activators have been used safely in patients [49].

## Results

### Cyclic Mechanical Stretch of Alveolar Epithelia Is Associated with a Transcriptional Program Resembling Hypoxia-Signaling

**Figure 1:**
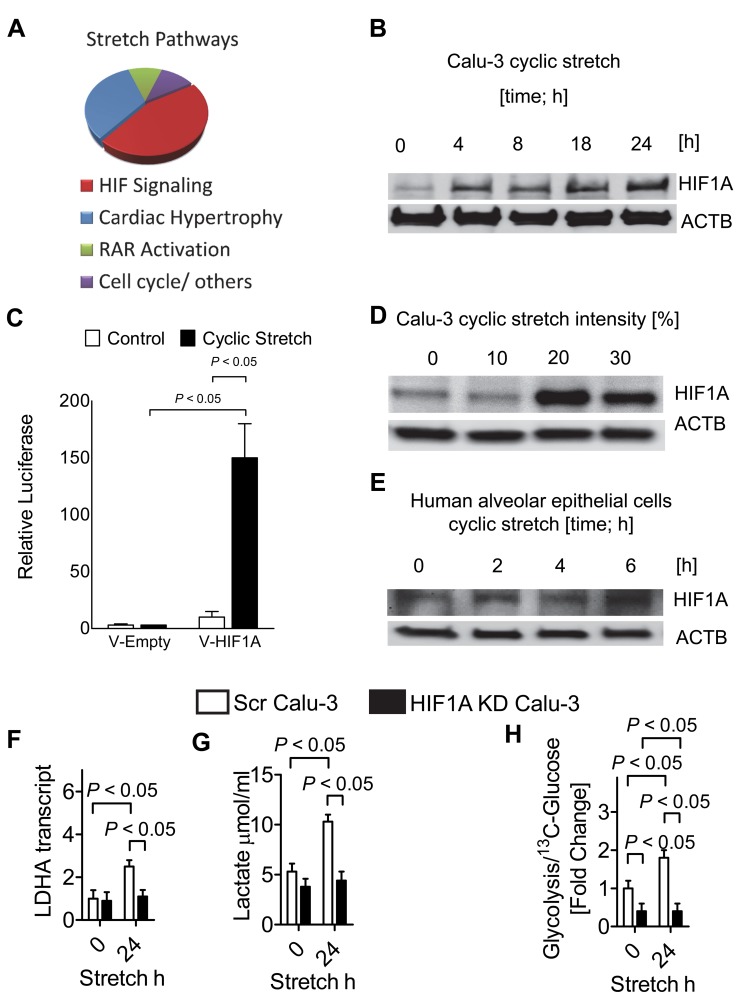
Alterations of gene-expression following cyclic mechanical stretch exposure of pulmonary epithelial cells—An in vitro model for ALI. Confluent Calu-3 or A549 cells were plated on collagen-coated BioFlex plates and underwent cyclic mechanical stretch for 24 h at 30% maximum, 0.7% stretch minimum, and sine wave 5 s on, 5 s off. (A) Pathway analysis. Microarray technology was used to assess transcriptional changes in stretch versus control conditions. (B) Calu-3 pulmonary epithelia exposed to different time periods of 30% stretch. HIF1A protein levels determined by Western blot. One representative blot of three is displayed. (**C**) HIF reporter plasmid consisting of four tandem HIF1A enhancer sequences from the 3′-region of the erythropoietin gene transfected into pulmonary epithelia (A549) and exposed to 30% stretch (*n* = 3). (D) Calu-3 pulmonary epithelia exposed to different degrees of cyclic mechanical stretch. HIF1A protein levels determined by Western blot. One representative blot of three is displayed. (E) Human alveolar epithelial cells (HPAEpiC) were exposed to different time-periods of stretch. Representative Western blots are shown following 30% stretch. (F) Transcript level of the glycolytic enzyme LDHA from HIF1A KD Calu-3 pulmonary epithelia (lentivirus mediated HIF1A knockdown, see Figure S2) or Calu-3 epithelia transduced with a control virus (lentiviral scrambled siRNA, Scr) after 24 h of stretch were determined by real-time RT-PCR relative to housekeeping gene beta-actin (mean ± s.d., *n* = 3). (G) Lactate levels in supernatants obtained from Calu-3 with a lentiviral-mediated HIF1A KD or controls (Scr) after 24 h of 30% stretch exposure. Controls consisted of Calu-3 cells treated with lentiviral scrambled siRNA exposed to the same experimental conditions (*n* = 4). (H) Calu-3 controls (Scr Calu-3; transfected with lentiviral scrambled siRNA) or Calu-3 HIF1KD were exposed to 24 h of 30% stretch with media containing the ^13^C-glucose isotope. For nuclear magnetic resonance (NMR) analysis of metabolites, cells were shock frozen immediately after stretch. Rate of glycolysis is given as ratio of incorporated ^13^C intermediates of glycolysis compared to ^13^C glucose levels (*n* = 4).

Previous studies had demonstrated that ALI is associated with significant alterations of gene-expression that frequently resembles endogenous adaptive responses [2],[7],[35]. To systematically examine alterations of pulmonary gene expression during ALI, we utilized a simple in vitro model of ALI by exposing human pulmonary epithelia to cyclic mechanical stretch conditions. This in vitro model resembles alveolar injury as occurs during ventilator-induced ALI in humans [22],[23],[40]. For this purpose, we examined human alveolar epithelial cells (Calu-3) following 24 h stretch exposure at 30% intensity by performing a genome microarray screen (http://www.ncbi.nlm.nih.gov/projects/geo/query/acc.cgi?acc=GSE27128). We subsequently confirmed the array results utilizing real-time RT-PCR and Western blotting for a subset of genes that were regulated (Table S1). Surprisingly, computerized pathway analysis to examine alterations in gene transcription (Ingenuity IPA, Version 11631407) revealed that hypoxia-signaling resembled the dominant stress response pathway when comparing stretch-exposed pulmonary epithelia to un-stretched controls (Figure 1A; Figure S1). In fact, subsequent studies of Calu-3 pulmonary epithelia exposed to different time-periods of stretch (Figure 1B) or studies utilizing a HIF reporter plasmid transfected into pulmonary epithelia (A549) and exposed to stretch conditions revealed stabilization of HIF1A—the key transcription factor for hypoxia adaptation (Figure 1C) [30]. Similarly, exposure to different degrees of cyclic mechanical stretch (Figure 1D) showed stretch-dose-dependent stabilization of HIF1A. Moreover, studies in primary human alveolar epithelial cells demonstrated robust HIF1A stabilization following stretch exposure (Figure 1E). To examine if HIF1A stabilization during stretch has functional consequences, we generated pulmonary epithelial cell lines with stable siRNA-mediated repression of HIF1A (Figure S2A–C). As HIF1A functions as an important regulator of glycolysis [50] we first explored the possibility of stretch controlling transcription of glycolytic enzymes. Exposure of control-transduced pulmonary epithelial cells demonstrated very robust induction of the transcript levels for phosphofructokinase m (PFKM), pyruvate dehydrogenase kinase 1 (PDK1) (Figure S3A), and lactate dehydrogenase a (LDHA, Figure 1F) with stretch exposure. In contrast, stretch-induced induction of glycolytic enzymes was completely abolished in pulmonary epithelial cells with HIF1A repression (Figure S3A and Figure 1F). Similarly, stretch-induced increases of lactate levels in their supernatant or increases in glycolytic flux as measured by metabolic turnover of ^13^C-labeled glucose during stretch were HIF1A-dependent (Figure 1G, H). These findings were specific for HIF1A, as cells with pulmonary epithelial cells with siRNA-mediated HIF2A repression behaved similar to controls (Figures S2 and S3). Together, these studies demonstrate stabilization of HIF1A during cyclic mechanical stretch exposure of human pulmonary epithelial cells in vitro and reveal transcriptional and functional consequences of HIF1A stabilization on carbohydrate metabolism.

### Stretch-Induced HIF1A Stabilization Occurs Under Normoxic Conditions and Requires Stretch-Elicited Inhibition of SDH

**Figure 2:**
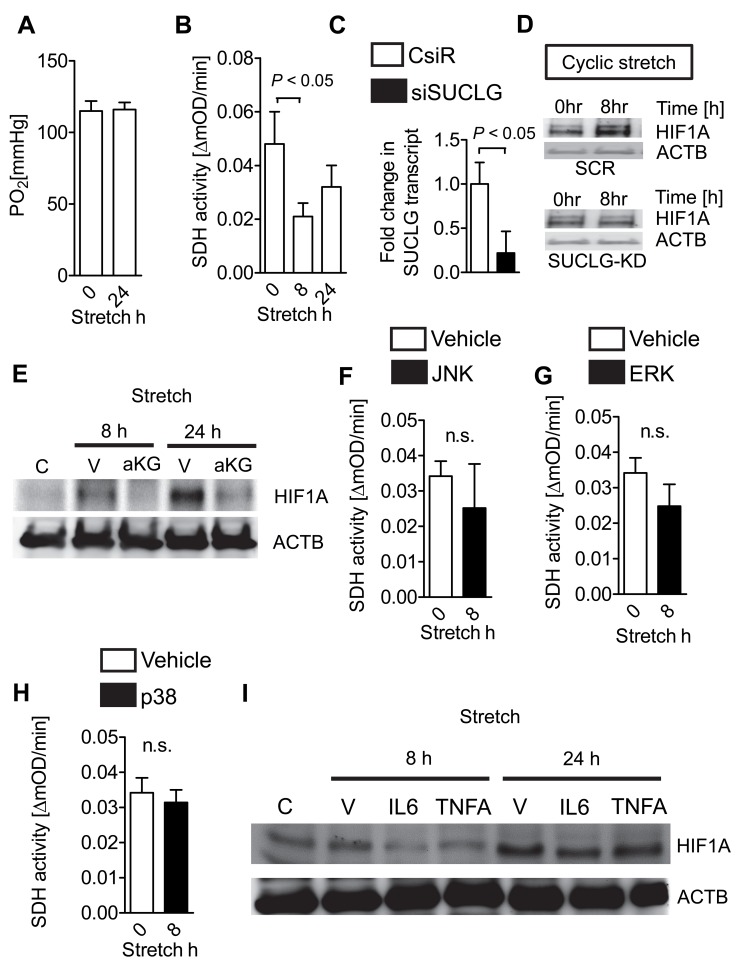
Mechanisms of cyclic mechanical stretch-mediated HIF1A stabilization. (A) Confluent Calu-3 cells underwent cyclic mechanical stretch for 24 h. Partial oxygen pressures (pO2) from supernatants were determined using I-STAT analyzer and compared to un-stretched controls (mean ± s.d., *n* = 3). (B) Following 8 or 24 h of cyclic mechanical stretch exposure, mitochondrial fractions of Calu-3 cells were obtained and analyzed for succinate dehydrogenase (SDH, mitochondrial Complex II) activity using ELISA. Activity is given as OD (optical density) change over time (mean ± s.d., *n* = 3). (C) siRNA knockdown of succinate-CoA ligase (SUCLG). RT PCR for SUCLG from A549 cells after siRNA treatment compared with control siRNA treatment (CsiR; beta-actin was used as house-keeping gene; *n* = 3). Note: siRNA knockdown revealed 82% reduction of SUCLG transcript. (D) A549 cells following siRNA repression of succinate-CoA ligase (siSUCLG) or treatment with nonspecific control siRNA (CsiR) were stretched for 0 or 8 h, lysed, and blotted for HIF1A. One representative blot of three is displayed. (E) Confluent Calu-3 cells underwent cyclic mechanical stretch for 8 or 24 h with and without α-ketoglutarate (aKG) treatment. Cells were lysed and blotted for HIF1A. One representative blot of three is displayed. (F–H) Confluent Calu-3 cells underwent cyclic mechanical stretch for 8 h with or without inhibitors of JNK, ERK, or p38. Mitochondrial fractions of Calu-3 cells were obtained and analyzed for succinate dehydrogenase (SDH, mitochondrial Complex II) activity using ELISA. (I) Confluent Calu-3 cells underwent cyclic mechanical stretch for 8 or 24 h with and without neutralizing antibodies for TNFA or IL-6. Cells were lysed and blotted for HIF1A. One representative blot of three is displayed.

Based on the above findings that identified stabilization and increased transcriptional and functional activity of HIF1A during cyclic mechanical stretch conditions, we next pursued studies to address the mechanism of stretch-induced HIF1A stabilization. As a first option, we considered the possibility that stretch-associated increases in oxygen consumption could lead to a decrease in oxygen availability, and subsequent hypoxic stabilization of HIF1A. Previous studies had shown that shifts in cellular oxygen levels between the cytosol and the mitochondria—as caused by nitric oxide—can cause HIF stabilization [51]. To address this possibility, we performed measurements of oxygen partial pressures within the supernatant of stretched pulmonary epithelial cells (Figure 2A) [52]. However, we observed high oxygen partial pressures in controls or stretched pulmonary epithelial cells, indicating that HIF1A stabilization during stretch occurs under normoxic conditions. Previous studies had indicated that normoxic stabilization of HIF in cancer involves inhibition or mutation of the metabolic enzyme SDH [46]. These studies demonstrate that mutations of SDH are associated with inhibition of prolylhydroxylases (PHDs)—a group of enzymes responsible for tagging HIF for proteasomal degradation [24],[53]–[55]—thereby causing normoxic HIF stabilization [46]–[48]. To examine the possibility that stretch-induced HIF1A stabilization could involve SDH inhibition, we next examined the consequences of pulmonary epithelial stretch exposure on SDH activity. We observed that stretch exposure was associated with a very robust attenuation of SDH activity (Figure 2B). To address if increased succinate levels with following PHD inhibition were the mechanism for HIF1A stabilization associated with low SDH activity, we generated cells with siRNA repression of succinate-CoA ligase (SUCLG; Figure 2C). While SDH inhibition leads to the accumulation of succinate and succinate-elicited PHD inhibition, SUCLG deletion would prevent the conversion of succinate-CoA to succinate and thereby lower succinate levels [46]–[48]. As shown in Figure 2D, stretch-induced HIF1A stabilization was completely abolished in cells with siRNA-mediated SUCLG repression, thereby implicating a “succinate leak”—caused by stretch-induced SDH inhibition—in normoxic HIF1A stabilization during cyclic mechanical stretch of human pulmonary epithelial cells.

To further address the possibility of stretch-elicited inhibition of SDH as a mechanism of normoxic HIF stabilization, we next performed studies with cell permeable α-ketoglutarate in studies on succinate-dependent HIF1A stabilization. Earlier studies found that succinate-mediated inhibition of PHD is competitive and is reversed by pharmacologically elevating intracellular α-ketoglutarate [56]. Introduction of α-ketoglutarate derivatives restores normal PHD activity and HIF1A levels and thereby alleviates pseudo-hypoxia. In fact and as shown in Figure 2E, α-ketoglutarate treatment prevented HIF1A stabilization in pulmonary epithelia at 8 and 24 h of stretch.

To address possible mechanisms of stretch/stress-induced SDH inhibition, we next pursued studies on the effect of stretch on stress kinases. Recent studies found enhanced mitogen-activated protein kinase (MAPK) activation following stretch of lung epithelia [57]. As such we measured SDH activity during stretch with and without inhibitors of JNK, ERK, or p38. As shown in Figure 2F–H, inhibition of these MAPKs abolished stretch-mediated SDH inhibition. Based on reports of normoxic HIF1A stabilization by pro-inflammatory cytokines [58], we next tested for stretch-induced HIF1A expression under conditions of neutralization of TNFA or IL-6 using neutralizing antibodies. As shown in Figure 2I, TNFA or IL-6 neutralizing antibodies blunted stretch-mediated HIF1A stabilization in lung epithelia. As TNFA is able to up-regulate stress kinases such as p38 [59], these findings indicate that TNFA release by stretched pulmonary epithelia might be involved in the up-regulation of stress kinases with concomitant inhibition of SDH activity.

**Figure 3:**
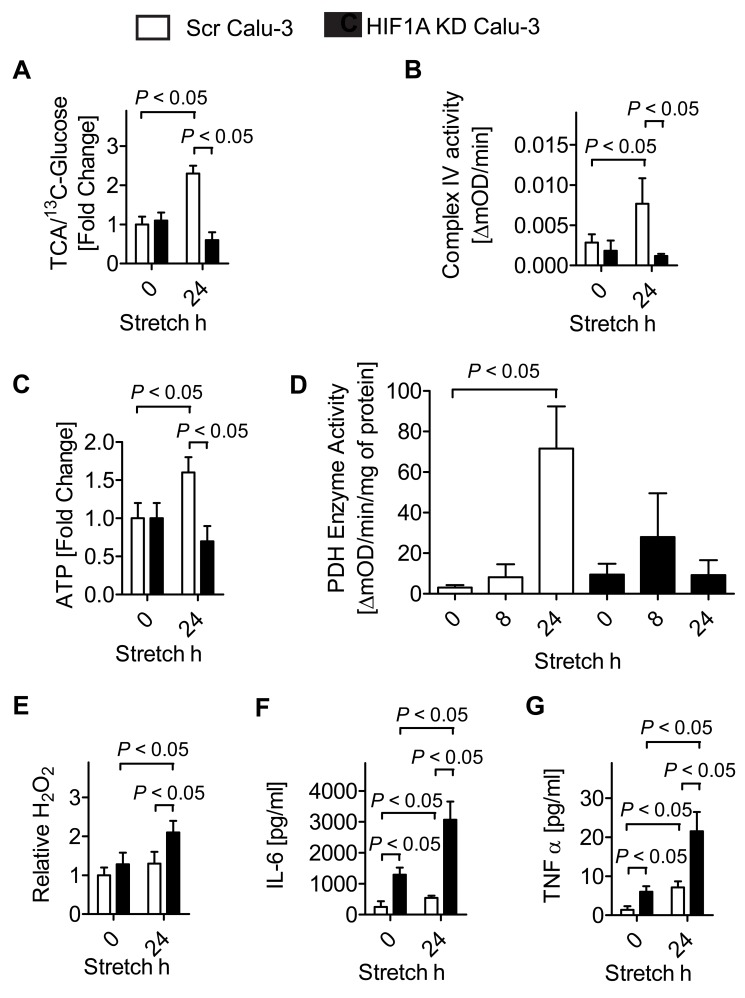
Functional consequences of cyclic mechanical stretch mediated HIF1A stabilization. (A) Calu-3 controls (Scr Calu-3; treated with lentiviral scrambled siRNA) or Calu-3 HIF1KD were exposed to 24 h of stretch with media containing the ^13^C-glucose isotope. For nuclear magnetic resonance (NMR) analysis of metabolites, cells were shock frozen immediately after stretch. Rate of tricarboxylic acid cycle (TCA) flux is given as ratio of incorporated ^13^C intermediates of the TCA compared to ^13^C glucose levels. (B) After 24 h of cyclic mechanical stretch, mitochondrial fractions of Calu-3 cells were obtained and analyzed for mitochondrial Complex IV (COX4) activity using ELISA. Activity is given as OD (optical density) change over time (mean ± s.d., *n* = 3). (C) ATP levels from Calu-3 controls (Scr Calu-3) or Calu-3 HIF1KD exposed to 24 of stretch. (D) Pyruvate dehydrogenase (PDH) activity levels from Calu-3 controls (Scr Calu-3) or Calu-3 HIF1KD exposed to 8 or 24 h of stretch. (E) Hydrogen peroxide levels of supernatants from Calu-3 controls or Calu-3 cells after 24 h at 30% stretch and 20% O2. The H_2_O_2_ data are expressed as the mean fluorescence levels from two independent experiments normalized by protein concentration. Error bars represents s.d. (*n* = 3). (F–G) IL-6 and TNF-α levels were evaluated in supernatants from stretched Calu-3 controls or Calu-3 HIF1KD exposed to 24 h of stretch using a human enzyme linked immunosorbent assay (ELISA). Results are presented as mean ± s.d. (*n* = 3).

To address the functional consequences of HIF on stretch-associated alterations of metabolism beyond anaerobic glycolysis, we next examined tricarboxylic acid cycle (TCA) flux and mitochondrial functions of human pulmonary epithelial cells (Calu-3) following siRNA-mediated HIF1A repression. As shown in Figure 3A, studies tracing different cellular metabolites in the presence of labeled glucose (^13^C-glucose) revealed that TCA flux was significantly increased in control cells, but not following siRNA-mediated HIF1A repression. As HIF1A functions to improve mitochondrial respiration and concomitant ATP production during hypoxia, we next assessed if HIF1A-dependent TCA flux increases during normoxia could be associated with increases of HIF1A-dependent mitochondrial complex IV (COX4) activity [60]. In fact, stretch exposure was associated with increased COX4 activity and ATP levels in controls, but not in pulmonary epithelial cells with HIF1A repression (Figure 3B,C). Together, these findings indicate that HIF1A optimizes metabolic functions during stretch conditions in vitro.

We next performed studies to better understand increases in TCA substrate flux despite up-regulation of PDK1. As shown, stretch stress causes normoxic HIF1A stabilization and HIF1A-dependent glycolytic enzymes expression. Among them pyruvate dehydrogenase kinase isoenzym 1 (PDK1) is up-regulated. This enzyme is not to be confused with phosphoinositide-dependent-kinase-1 (also abbreviated PDK1). The pyruvate dehydrogenase kinase isoenzym 1 (PDK1) phosphorylates the enzyme of the same name (specifically PDE1), which is the major component of the pyruvate dehydrogenase complex (PDC). Phosphorylation of pyruvate dehydrogenase at serine residue 1 of 3 possible ones by PDK1 will almost completely inhibit activity of the PDC. As shown in Figure 1G, inhibition of the PDC fits well with increased production of lactate, as pyruvate is expected to accumulate because it is less metabolized in the first step of the TCA controlled by PDC activity. However, how does up-regulation of PDK1, a master inhibitor of the PDC, fit to a several-fold increase in TCA/C13-Gucose ratios (Figure 2E). To address this question, we next measured mitochondrial PDC activity [61] during stretch conditions. In particular, we measured the activity of the pyruvate dehydrogenase (PDH). As shown in Figure 3D we found increases of PDH activity in a HIF1A-dependent manner upon stretch. These findings further supports our hypothesis of normoxic HIF1A stabilization and HIF1A-dependent increases of TCA cycle activity despite findings of PDK1 mRNA induction (Figure S3). Consistent with a mitochondrial dysfunction in HIF1A knock-down cells, we next observed increased reactive oxygen species (H_2_O_2_, Figure 3E). Moreover, markers for epithelial cell inflammation were elevated in Calu-3 cells with HIF1A repression (Figure 3F–G). Similarly to the metabolic alterations, these findings were specific for HIF1A, since HIF2A knockdown cells showed similar responses as controls (Figure S3C). Together, these findings indicate a functional role for normoxic HIF1A stabilization during conditions of cyclic mechanical stretch in attenuating pulmonary epithelial inflammation by optimizing cellular metabolism of carbohydrates.

### Ventilator-Induced ALI Is Associated with Normoxic HIF1A Stabilization in Vivo

**Figure 4:**
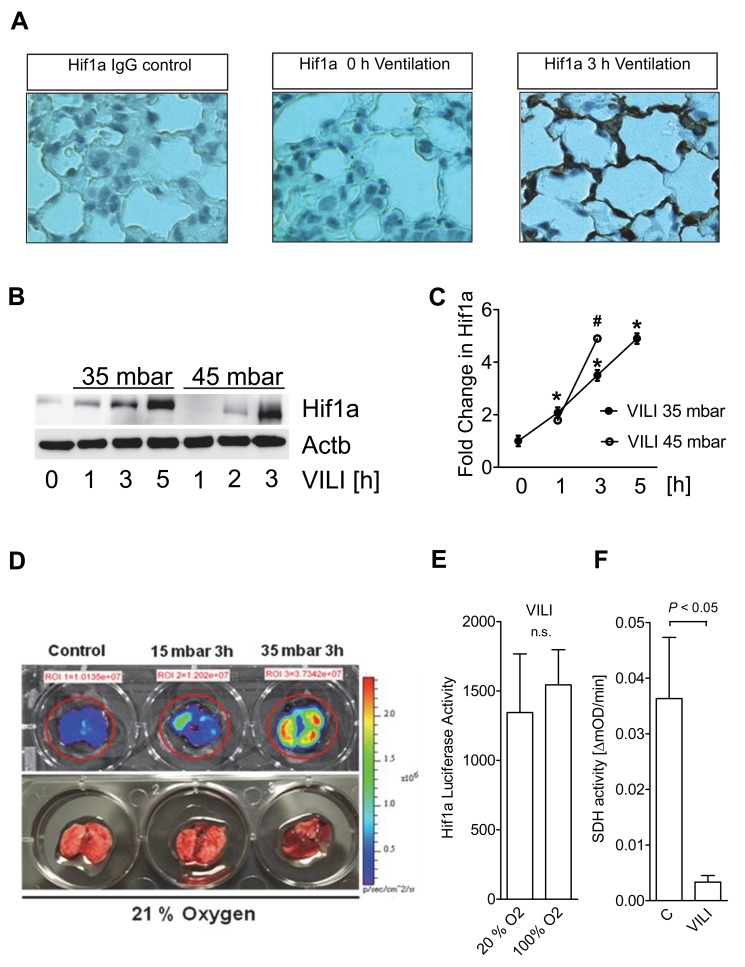
Normoxic HIF1A stabilization during ALI. (**A**) Wild-type mice (BL6C57) were exposed to ventilator induced lung injury (VILI; pressure-controlled mechanical ventilation at an inspiratory pressure of 45 mbar with an inspired oxygen concentration of 100%, exposure time 180 minutes). To examine the influence of mechanical ventilation on pulmonary HIF1A expression patterns, lungs were stained with antibodies for Hif1a. IgG controls were used at identical concentrations and staining conditions as the target primary antibodies (magnification ×400; *n* = 4). (B) Frozen lung tissue was lysed and proteins resolved by SDS-PAGE. Resultant Western blots were probed with anti-Hif1a antibody. A representative blot of three is displayed. (C) Quantitative analysis of Western blot in (B) assessed by densitometry. (D) Imaging HIF1A using previously described HIF reporter mice expressing luciferase upon HIF stabilization (“ODD-Luc mice”) during ALI. Prior to imaging, mice were injected with i.p. luciferin (50 mg/kg) and mice were euthanized. Left column (control): Lungs excised without mechanical ventilation. To induce ALI, mice were ventilated with pressure controlled ventilation (45 mbar) at 21% oxygen concentration over 3 h. Middle column, 3 h at 15 mbar; right column, 3 h at 35 mbar. Color bar indicates photons/(cm2·s·steradian) with minimum and maximum threshold values (*n* = 4). (E) “ODD-Luc mice” were exposed to VILI (45 mbar, 3 h) using 20% or 100% inspired oxygen. Lungs were harvested, homogenized, and analyzed for luciferase gene expression using the Dual-Luciferase Reporter Assay System from Promega. (F) After 3 h of ventilation at 45 mbar, mitochondrial fractions obtained from lung tissue were analyzed for succinate dehydrogenase (SDH, mitochondrial Complex II) activity using ELISA. Activity is given as OD (optical density) change over time (mean ± s.d., *n* = 3).

After having observed a transcriptional hypoxia-program in pulmonary epithelia exposed to stretch conditions in vitro, we went on to examine HIF1A during in vivo conditions of ALI. For this purpose, we utilized a previously described model of mechanical ventilation-induced ALI [23],[40],[62]. We gained initial insight from immune-histochemical studies of pulmonary tissues following exposure to ALI. Consistent with in vitro studies of stretch exposure, we observed increased HIF1A staining following ALI induction (Figure 4A). Similarly, Western blotting indicated that HIF1A stabilization during ALI is time-dependent and occurs more pronounced with increased stretch conditions—such as those encountered during mechanical ventilation at higher inspiratory pressure levels (35 mbar versus 45 mbar; Figure 4B,C). In addition, we examined pulmonary HIF1A levels utilizing a previously characterized HIF reporter mouse model [63]. Consistent with the above studies utilizing Western blotting for HIF1A, we observed elevated HIF1A levels following ALI induction (Figure 4D). Interestingly, studies utilizing different concentrations of oxygen in the inspired air during ventilator-induced ALI (20% oxygen versus 100% oxygen) demonstrated that HIF1A stabilization is not affected by increasing the level of inspired oxygen concentration—suggesting that HIF1A stabilization occurs under conditions with sufficient oxygen availability (Figure 4E). Similarly to stretch-associated HIF stabilization, we observed robust repression of SDH activity following exposure to ALI induced by mechanical ventilation (Figure 4F), thereby implicating SDH-associated increases in succinate in the normoxic stabilization of HIF during ALI. Together, these findings reveal normoxic stabilization of HIF during ventilator-induced ALI.

### Pharmacologic HIF1A Stabilization Attenuates Lung Inflammation and Pulmonary Edema During ALI

**Figure 5:**
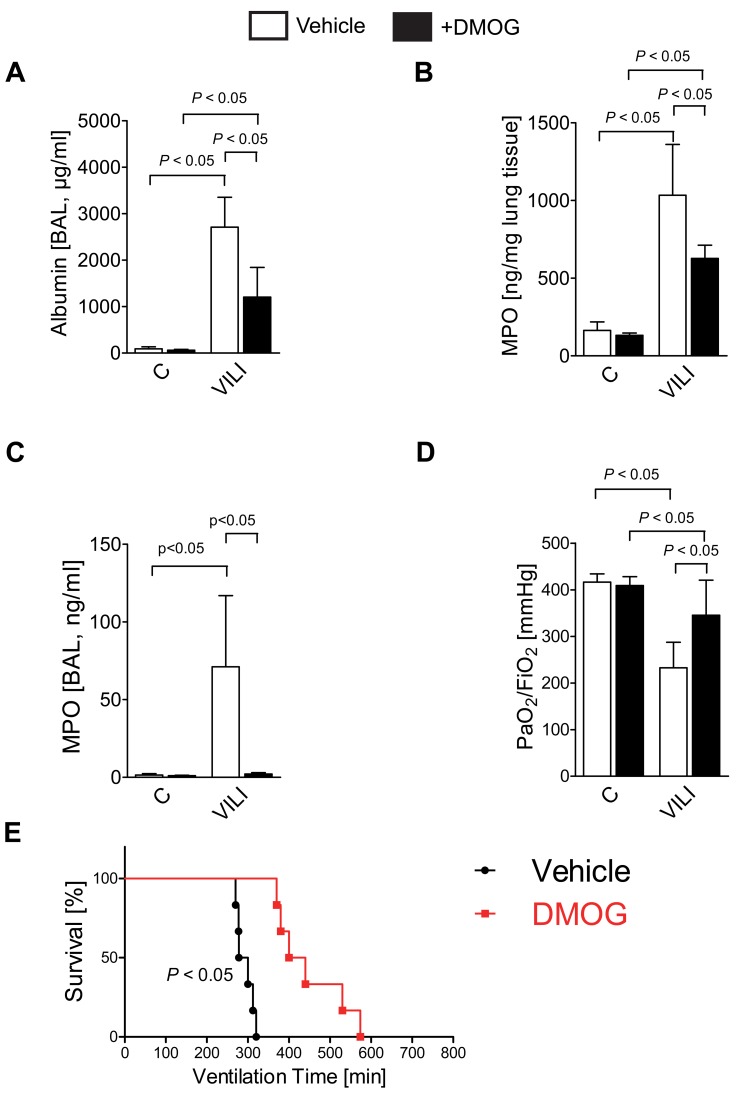
Functional consequences of HIF1A activation during ALI. (A–E) HIF1A activator dimethyl-oxaloylglycine (DMOG) during ALI: BL6C57 mice were treated with 1 mg DMOG or vehicle control 4 h prior to the experimental procedure. (A) Mechanical ventilation was instituted and mice were ventilated for 0 or 180 min using pressure-controlled settings (inspiratory pressure of 45 mbar, 100% inspired oxygen concentration). Albumin concentration in the bronchoalveolar fluid (BAL) was determined by enzyme linked immunosorbent assay (*n* = 6). (B and C) Pulmonary neutrophil sequestration was quantified using a myeloperoxidase (MPO) assay. MPO activity was assessed using a murine ELISA from lung tissue (B) or BAL (C) (mean ± s.d., *n* = 6). (D) To assess pulmonary gas exchange, blood gas analyses were performed by obtaining arterial blood via cardiac puncture. Analysis was performed immediately and the ratio of the arterial partial pressure of oxygen (PaO2) to the fraction of inspired oxygen (FiO2) was determined (mean ± s.d., *n* = 6). (E) Mechanical ventilation was instituted and mice were ventilated using pressure-controlled settings (inspiratory pressure of 35 mbar, 100% inspired oxygen concentration) until a cardiac standstill was observed in the surface electrocardiogram (*n* = 6).

Based on the above finding that HIF1A is stabilized during ALI, we next performed pharmacologic studies to address the functional role of HIF1A stabilization on ALI outcomes. First, we utilized the HIF1A activator dimethyl-oxaloylglycine (DMOG). DMOG-associated stabilization of HIF1A involves inhibition of PHDs [64],[65]—a mechanism very similar to succinate-dependent HIF stabilization. For the purpose of these studies, we pretreated mice with DMOG (1 mg i.p.) 4 h prior to the induction of ALI. This pharmacologic approach was associated with robust HIF1A stabilization in murine lungs (Figure S4A). Functional studies revealed that ALI-associated increases in pulmonary edema were dramatically improved following DMOG treatment (Figure 5A). In addition, pre-treatment with DMOG was associated with attenuated lung inflammation, as assessed by measurements of myeloperoxidase levels in the lungs or in the BAL, indicating that pretreatment with DMOG attenuates pulmonary neutrophil accumulation in the lungs during ALI (Figure 5B,C). Similarly, attenuated gas exchange during VILI was improved in mice pre-treated with DMOG (Figure 5D). Finally, pre-treatment with DMOG was also associated with a very dramatic increase of survival time during VILI exposure (Figure 5E). Together, these pharmacologic studies indicate that stabilization of HIF during ALI functions to dampen pulmonary edema and lung inflammation during ALI induced by mechanical ventilation in vivo.

### Inhibition of HIF1A Functions Is Detrimental During ALI

**Figure 6:**
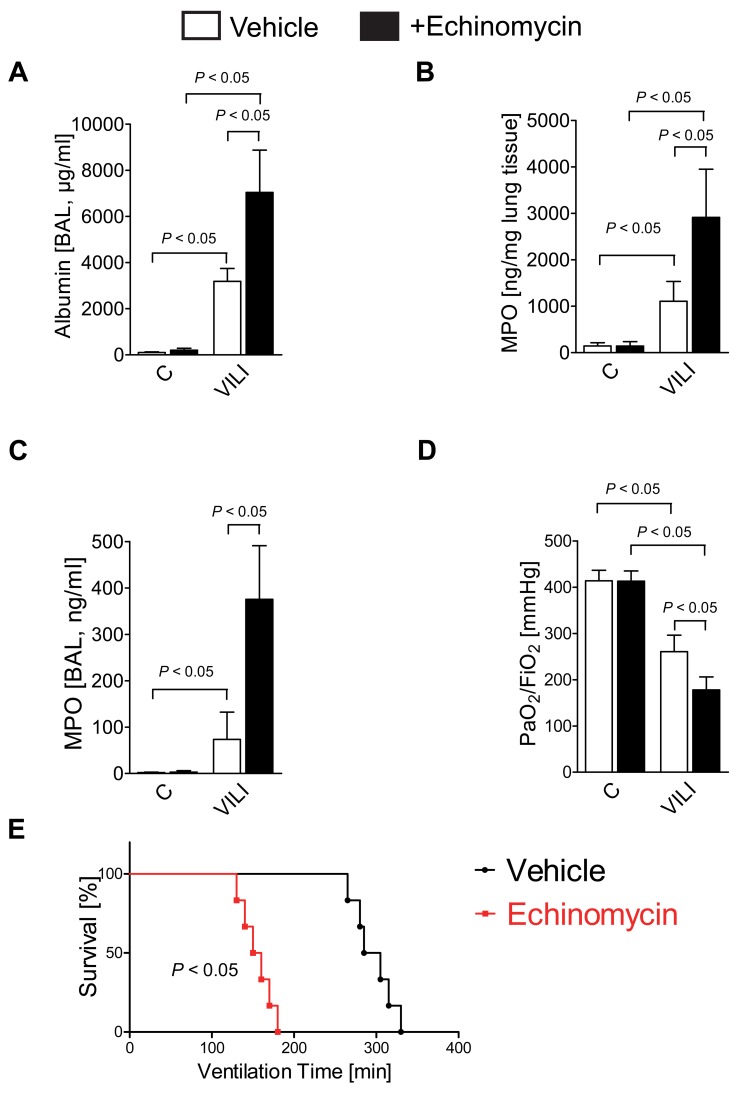
Functional consequences of HIF1A inhibition during ALI. (A–E) HIF1A inhibition by Echinomycin during VILI: BL6C57 mice were treated with 30 µg Echinomycin i.p. or vehicle control 1 h prior to the experimental procedure. (A) Mechanical ventilation was instituted and mice were ventilated for 0 or 180 min using pressure-controlled settings (pressure-controlled mechanical ventilation at an inspiratory pressure of 45 mbar with an inspired oxygen concentration of 100%, exposure time 180 min). Albumin concentration in the bronchoalveolar fluid (BAL) was determined by enzyme linked immunosorbent assay (*n* = 6). (B and C) Pulmonary neutrophil sequestration was quantified using a myeloperoxidase (MPO) assay. MPO activity was assessed using a murine ELISA from lung tissue (B) or BAL (C) (*n* = 6). Results are presented as mean ± s.d. and derived from six animals in each condition. (D) To assess pulmonary gas exchange, blood gas analyses were performed by obtaining arterial blood via cardiac puncture. Analysis was performed immediately and the ratio of the arterial partial pressure of oxygen (PaO2) to the fraction of inspired oxygen (FiO2) was determined (mean ± s.d., *n* = 6). (E) Mechanical ventilation was instituted and mice were ventilated using pressure-controlled settings (inspiratory pressure of 35 mbar, 100% inspired oxygen concentration) until a cardiac standstill was observed in the surface electrocardiogram (*n* = 6).

After having observed very robust therapeutic effects for the treatment with a pharmacologic HIF activator, we next examined the consequences of a pharmacologic inhibitor of HIF1A activity. For these studies we utilized echinomycin, which prevents binding of HIF1A to the DNA, thereby preventing functional HIF1A activation [66]. Echinomycin pretreatment prevented HIF-dependent induction of pulmonary HIF target genes, such as PFKM, PDK1, or LDHA transcript levels (Figure S4B). In contrast to studies with the HIF1A activator DMOG, we observed increased pulmonary edema (Figure 6A), in conjunction with increased pulmonary and BAL myeloperoxidase levels (Figure 6B,C), attenuated gas exchange (Figure 6D), and decreased survival time during ventilator-induced ALI in mice that were pre-treated with the HIF inhibitor echinomycin (Figure 6E). Together, these pharmacologic studies indicate that inhibition of the transcriptional functions of HIF during ALI is highly detrimental, implicating normoxic HIF1A stabilization in an endogenous feedback loop to protect the lungs from excessive inflammation and pulmonary edema during ALI.

### Alveolar-Epithelial HIF Signaling Mediates Lung Protection During ALI

**Figure 7:**
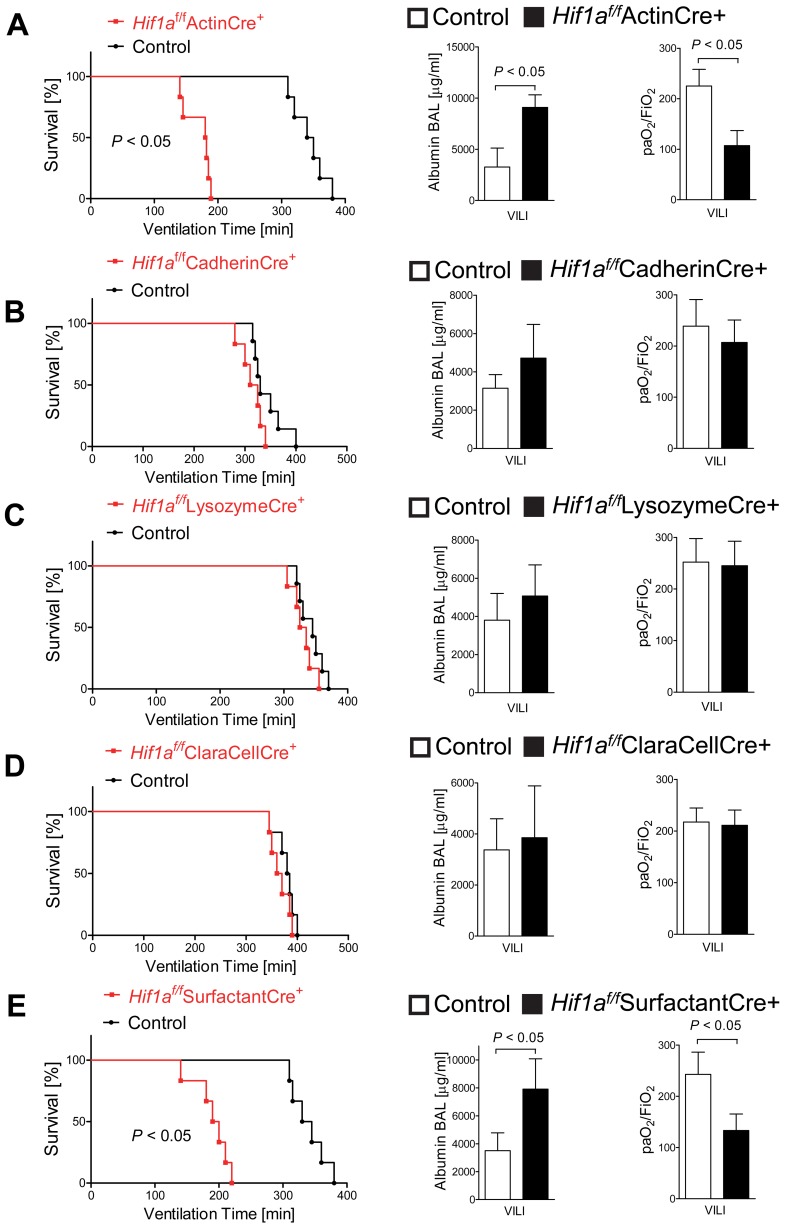
Tissue-specific function of HIF1A during ALI. *Hif1a^f/f^* Actin-Cre+ mice (A), *Hif1a^f/f^* CadherinCre+ (B), *Hif1a^f/f^* LysozymCre+ (C), *Hif1a^f/f^* ClaraCellCre+ (D), or *Hif1a^f/f^* SurfactantCre+ (E) or corresponding age, weight, and gender-matched littermate controls (Cre expressing mice) were exposed to ventilator-induced lung injury (VILI; pressure-controlled mechanical ventilation at an inspiratory pressure of 35 mbar with an inspired oxygen concentration of 100%). Survival time was determined by measuring the time until a cardiac standstill was observed in the surface electrocardiogram. Note significantly attenuated survival of *Hif1a^f/f^* Actin-Cre+ mice or *Hif1a^f/f^* SurfactantCre+ (A, E, mean ± s.d., *p*<0.05, *n* = 6). Albumin concentration in the bronchoalveolar fluid was determined by enzyme-linked immunosorbent assay (ELISA; pressure-controlled mechanical ventilation at an inspiratory pressure of 45 mbar with an inspired oxygen concentration of 100%, exposure time 180 min). Note significantly increased albumin concentration in the bronchoalveolar fluid of *Hif1a^f/f^* Actin-Cre+ mice or *Hif1a^f/f^* SurfactantCre+ (A, E, mean ± s.d., *p*<0.05, *n* = 6).

To gain insight into the tissue-specific source of HIF1A-dependent lung protection, we next performed genetic studies for HIF1A during ventilator-induced ALI. Due to the fact that *Hif1a* ^−/−^ mice die during embryogenesis [67]–[69], we utilized transgenic mice with a “floxed” HIF1A gene [70] to systematically delete HIF1A in different tissue compartments of the lungs. As first step, we generated mice with induced deletion of HIF1A in all tissues, including the lungs (*Hif1a^f/f^*ActinCre+, Figure S4C). Consistent with our above findings with pharmacologic HIF inhibition, we observed dramatic decreases in survival time, paired with increased pulmonary edema and attenuated gas exchange during ALI exposure of *Hif1a^f/f^* ActinCre+ mice compared to ActinCre+ controls (Figure 7A). In contrast, mice with deletion of HIF1A in vascular endothelia (*Hif1a^f/f^* CadherinCre+; Figure 7B, Figure S5A), myeloid cells (*Hif1a^f/f^* LysozymCre+; Figure 7C) [70], or the conducting airways (*Hif1a^f/f^* ClaraCellCre+; Figure 7D, Figure S5B) [71] showed no difference in survival time, pulmonary edema, or gas-exchange during ALI as compared to corresponding controls. However, mice with induced deletion of HIF1A in their alveolar epithelial cells (*Hif1a^f/f^* SurfactantCre+; Figure 7E, Figures S5C and S6) [72] showed a similar phenotype as mice with induced deletion of HIF in all tissues. Indeed, *Hif1a^f/f^* SurfactantCre+ demonstrated attenuated survival time, increased pulmonary edema, and attenuated gas exchange during ventilator-induced ALI. Taken together, these studies provide genetic in vivo evidence for a protective role for alveolar-epithelial-specific HIF1A-signaling during ALI.

### *Hif1a^f/f^* SurfactantCre+ Mice Experience Profound Lung Inflammation During ALI

**Figure 8:**
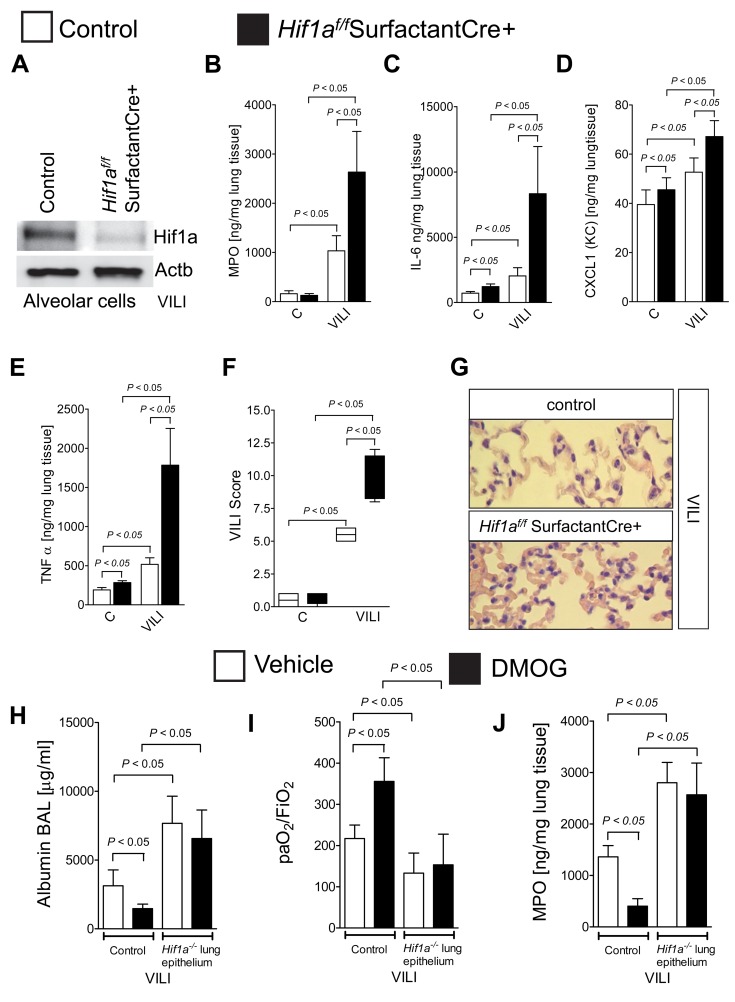
Functional consequences of alveolar HIF1A deletion during ALI. (A–J) *Hif1a^f/f^* SurfactantCre+ mice or age, gender, and weight-matched littermate controls (SurfactantCre+) were exposed to ventilator-induced lung injury (VILI; pressure-controlled mechanical ventilation at an inspiratory pressure of 45 mbar with an inspired oxygen concentration of 100%, exposure time 180 min). (A) Isolation of alveolar epithelial cells from *Hif1a^f/f^* SurfactantCre+ or control mice after VILI exposure. Frozen cells were lysed and proteins resolved by SDS-PAGE. Resultant Western blots were probed with anti-Hif1a antibody. A representative blot of three is displayed. (B) Pulmonary neutrophil accumulation was quantified using myeloperoxidase (MPO) ELISA. (C–E) IL-6, KC, or TNF-α, levels were evaluated in lung tissue homogenates using a mouse-enzyme-linked immunosorbent assay (ELISA). Results are presented as mean ± s.d. (*n* = 6). (F) For quantification of histological tissue damage by ventilator-induced lung injury following 180 min ventilation, VILI scores were assessed in *Hif1a^f/f^* SurfactantCre+ or corresponding littermate control mice (SurfactantCre+). Results are displayed as median and range (*n* = 4). (G) One of four representative photomicrographs (×200) stained with hematoxylin/eosin is displayed. (H–J) *Hif1a^f/f^* SurfactantCre+ or matched controls (Surfactant Cre+) were treated with the pharmacologic HIF activator dimethyl-oxaloylglycine DMOG (1 mg/mouse i.p. 4 h period to VILI) and subsequently exposed to VILI. (H) Albumin concentration in the bronchoalveolar fluid by enzyme-linked immunosorbent assay (ELISA), (I) pulmonary gas exchange by the ratio of the arterial partial pressure of oxygen (PaO2) to the fraction of inspired oxygen (FiO2), and (J) MPO activity by using a murine ELISA from lung tissue (mean ± s.d.; *n* = 6).

Based on the above studies implicating alveolar epithelial HIF1A in lung protection during ALI, we performed a more detailed examination of mice with conditional deletion of HIF1A in pulmonary epithelia (*Hif1a^f/f^* SurfactantCre+). Isolation of alveolar epithelial cells from *Hif1a^f/f^* SurfactantCre+ or control mice after exposure to mechanical ventilation confirmed alveolar-epithelial HIF1A stabilization in controls but not in *Hif1a^f/f^* SurfactantCre+ (Figure 8A, Figure S6). Additional functional studies of *Hif1a^f/f^* SurfactantCre+ demonstrated a more profound degree of lung inflammation during ALI, including increased pulmonary neutrophil accumulation (Figure 8B), pulmonary IL-6 (Figure 8C), CXCL1 (Figure 8D), and pulmonary TNF-α levels (Figure 8E). Similarly, pulmonary injury as assessed by a VILI score (Figure 8F) and as shown in a representative histologic slides (Figure 8G) was dramatically increased in *Hif1a^f/f^* SurfactantCre+ upon exposure to ALI induced by mechanical ventilation. In addition, the lung protective effects of pharmacologic treatment with the HIF activator DMOG on albumin leakage (Figure 8H), pulmonary gas exchange (Figure 8I), and neutrophil accumulation in the lungs (Figure 8J) were abolished in mice with conditional HIF1A deletion in the alveolar epithelia, indicating that DMOG-elicited lung protection requires the presence of pulmonary epithelial HIF1A, as opposed to alternative tissue-specific sources for HIF1A such as endothelia or myeloid cells. Taken together, these findings implicate alveolar-epithelial HIF1A in dampening lung inflammation during ALI.

### Alveolar-Epithelial HIF1A Dampens Lung Inflammation by Enhancing Glycolysis and Optimizing Mitochondrial Respiration During ALI

**Figure 9:**
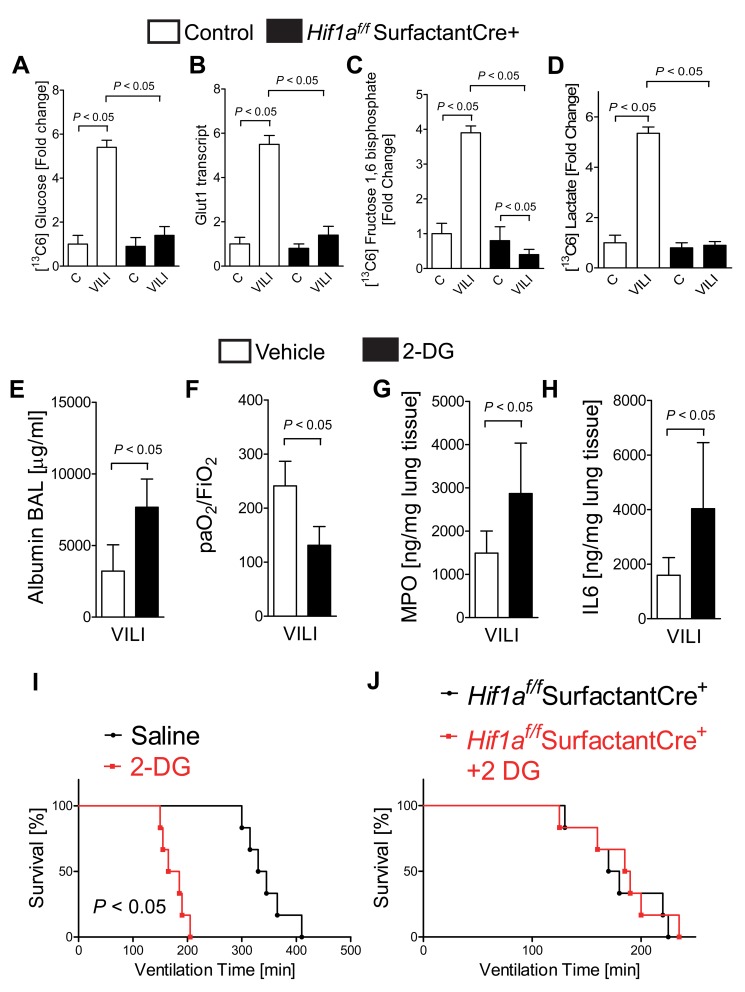
Functional role of alveolar epithelial HIF1A on glycolysis during ALI. (A–D) *Hif1a^f/f^* SurfactantCre+ mice or littermate controls (SurfactantCre+) matched in age, weight, and gender were exposed to ventilator-induced lung injury (VILI; pressure-controlled mechanical ventilation at an inspiratory pressure of 45 mbar with an inspired oxygen concentration of 100%, exposure time 120 min). ^13^C glucose was administered i.p. 30 min prior to the experimental procedure. Determination of ^13^C glucose and ^13^C carbohydrates during VILI was performed using liquid chromatography–tandem mass spectrometry (LC-MS). Glut 1 transcript level was determined by real-time RT-PCR relative to house-keeping gene beta-actin and expressed as fold induction relative to sham-operated controls (mean ± SD, *n* = 3). (A) ^13^C glucose. (B) Glut-1 transcript levels. (C) ^13^C fructose 1,6 bisphosphate. (D) ^13^C lactate. (E–H) To examine the functional role of glycolysis, wild-type mice treated with the glycolysis inhibitor 2-deoxy-D-glucose (200 mg/kg dose, i.p.) 30 min prior to the experimental procedure, and subsequently exposed to VILI (see above). Albumin concentration in the bronchoalveolar fluid (E) by enzyme-linked immunosorbent assay (ELISA), pulmonary gas exchange (F) by the ratio of the arterial partial pressure of oxygen (PaO2) to the fraction of inspired oxygen (FiO2), and MPO activity by using a murine ELISA from lung tissue (G) or IL-6 levels (H) in lung tissue homogenates using a mouse enzyme-linked immunosorbent assay (ELISA). Results are presented as mean ± s.d. (*n* = 6). (I and J) Mechanical ventilation was instituted and SurfactantCre+ controls or *Hif1a^f/f^* SurfactantCre+ mice with and without 2-DG treatment were exposed to VILI (pressure-controlled mechanical ventilation at an inspiratory pressure of 35 mbar with an inspired oxygen concentration of 100%) until a cardiac standstill was observed in the surface electrocardiogram (*p*<0.05, *n* = 6).

Based on the above in vitro studies on the role of HIF1A in optimizing pulmonary epithelial carbohydrate metabolism during conditions of cyclic mechanical stretch, we next examined metabolic functions of alveolar-epithelial HIF1A during ALI in vivo. For this purpose—and as we have done in previous studies [73]—we employed liquid chromatography–tandem mass spectrometry analysis of ^13^C-glucose metabolites. In line with the hypothesis that alveolar-epithelial HIF1A optimizes carbohydrate metabolism during ALI in vivo, we found that elevations of pulmonary glucose levels during ALI were completely abolished in *Hif1a^f/f^* SurfactantCre+ (Figure 9A). Similarly, transcript levels of the glucose transporter Glut1 were dramatically increased following ALI in control mice but not in *Hif1a^f/f^* SurfactantCre+ (Figure 9B). Moreover, we observed that ALI-associated elevations of [^13^C6]-fructose 1,6 bisphosphate and [^13^C6]-lactate following exposure to ALI were completely abolished in *Hif1a^f/f^* SurfactantCre+ (Figure 9C,D). Similarly, ALI-associated elevations of the transcript levels of glycolytic enzymes were attenuated in *Hif1a^f/f^* SurfactantCre+ mice (Figure S7A). Taken together, these data indicate that ALI induced by mechanical ventilation is associated with a transcriptional program under the control of alveolar-epithelial HIF1A that leads to an increase in glycolytic flux. To address the functional role of HIF-dependent induction of the glycolytic pathway, we next performed in vivo studies with the glycolysis inhibitor 2-deoxy-D-glucose (2-DG), a well-characterized inhibitor of glycolysis [74]. Here we found that treatment with 2-DG (200 mg/kg i.p. prior to the induction of ventilator-induced ALI) was associated with increased pulmonary edema (Figure 9E), attenuated gas exchange (Figure 9F), enhanced pulmonary neutrophil accumulation as assessed by MPO levels (Figure 9G), and cytokine production (IL-6; Figure 9H). Moreover, survival time during ALI exposure of anesthetized mice was significantly reduced following pretreatment with 2-DG as compared to vehicle treatment in wild-type mice (Figure 9I), while 2-DG treatment of *Hif1a^f/f^* SurfactantCre+ mice had no effect on survival time (Figure 9J). Similar, while 2-DG treatment increased H_2_O_2_ production in wild-type mice—indicating mitochondrial stress—2-DG treatment did not further increase H_2_O_2_ levels in *Hif1a^f/f^* SurfactantCre+ mice (Figure S7B). Taken together, these studies indicate that enhancing the glycolytic carbohydrate flux during ALI is mediated exclusively through alveolar epithelial HIF1A. Moreover, pharmacologic studies using an inhibitor for glycolysis implicate transcriptional increases in lung epithelial glucose metabolism as an endogenous adaptive pathway to dampen inflammation during ALI in vivo.

### Alveolar Epithelial HIF1A Increases TCA Flux and Mitochondrial Activity During ALI in Vivo

**Figure 10:**
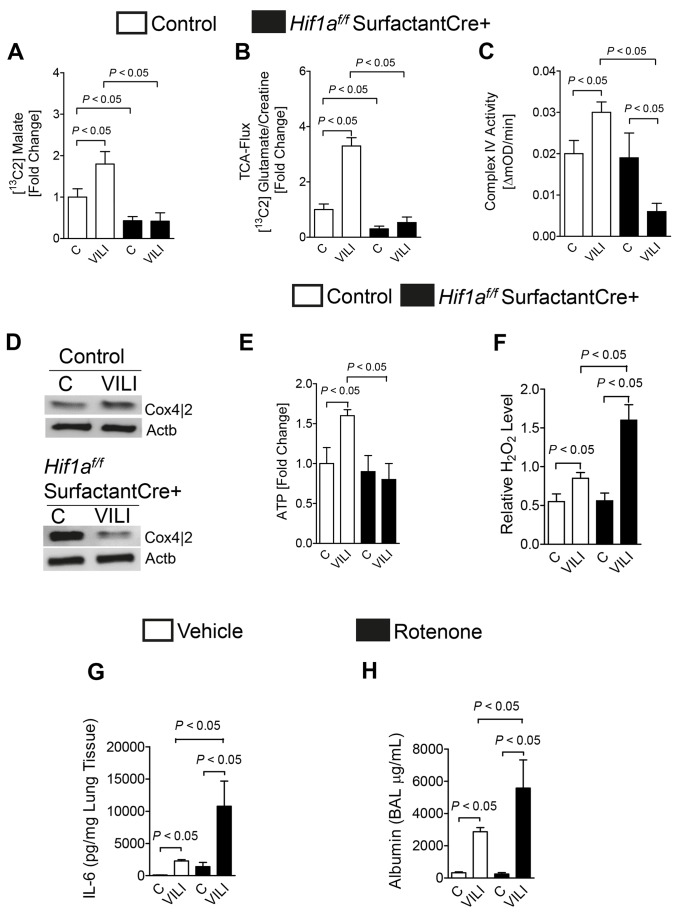
Consequences of alveolar-epithelial Hif1a deletion on mitochondrial function during ALI. (A–G) *Hif1a^f/f^* SurfactantCre+ mice or littermate controls (SurfactantCre+) matched in age, weight, and gender were exposed to ventilator-induced lung injury (VILI; pressure-controlled mechanical ventilation at an inspiratory pressure of 45 mbar with an inspired oxygen concentration of 100%, exposure time 120 min). ^13^C glucose was administered i.p. 30 min prior to the experimental procedure. Determination of ^13^C carbohydrates during VILI was performed using liquid chromatography–tandem mass spectrometry (LC-MS). (A) ^13^C malate. (B) Tricarboxylic acid (TCA) cycle flux rates were determined by measuring the ratio of ^13^C2 glutamate to total creatine (*n* = 4 for all experiments). (C–F) *Hif1a^f/f^* SurfactantCre+ mice or age-, gender-, and weight-matched littermate controls (SurfactantCre+) were exposed to VILI (see above). (C) After 3 h of VILI exposure, mitochondrial fractions were obtained from lung tissue and analyzed for Complex IV activity using ELISA. Activity is given as OD (optical density) change over time (mean ± s.d., *n* = 3). (D) Frozen lung tissue was lysed and mitochondrial proteins resolved by SDS-PAGE. Resultant Western blots were probed with anti-COX4-2 antibody. A representative blot of three is shown. (E) Pulmonary ATP content in *Hif1a^f/f^* SurfactantCre+ mice or littermate controls (SurfactantCre+) were exposed to VILI (inspired oxygen concentration 100%, exposure time 120 min, pressure-controlled ventilation with an inspiratory pressure of 45 mbar). (F) Hydrogen peroxide lung tissue levels in *Hif1a^f/f^* SurfactantCre+ mice or littermate controls (SurfactantCre+) exposed to VILI. Data are expressed as the mean fluorescence levels from three independent experiments normalized by protein concentration. Error bars represent s.d. (*n* = 3). (G and H) BL6C57 mice were treated with Rotenone—an inhibitor of mitochondrial respiration—or vehicle control 1 h prior to the beginning of the experimental procedure. IL-6 levels in lung tissue homogenates (G) or Albumin levels in BAL (H) were evaluated using a mouse enzyme-linked immunosorbent assay (ELISA) following exposure to VILI (see above). Results are presented as mean ± s.d. (*n* = 6).

**Figure 11:**
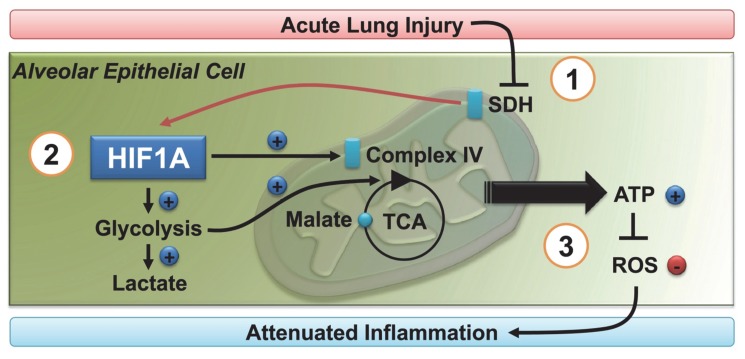
Proposed model of ALI-associated HIF1A stabilization. (*1*) During ALI, stretch of pulmonary epithelial cells results in the inhibition of succinate dehydrogenase (SDH), leading to normoxic stabilization of alveolar epithelial HIF1A. (*2*) Alveolar epithelial HIF1A results in increased glycolytic capacity, TCA flux, and optimized mitochondrial respiration via induction of Complex IV. (*3*) HIF1A-dependent prevention of mitochondrial dysfunction during ALI is associated with increased alveolar epithelial capacity to produce ATP, while concomitantly preventing ROS accumulation and attenuating lung inflammation.

To gain additional insight into the metabolic functions of epithelial HIF1A during ALI in vivo, we next pursued metabolic studies on TCA flux and mitochondrial metabolism in *Hif1a^f/f^* SurfactantCre+ mice. Consistent with the above studies in stretched pulmonary epithelial cells in vitro, we observed that ALI-associated increases in [^13^C]-malate and TCA flux rates were completely abolished in mice with gene-targeted deletion of HIF1A in alveolar epithelia (Figure 10A,B), suggesting that alveolar epithelial HIF1A specifically promotes TCA flux during ALI in vivo. Based on our in vitro studies implicating HIF1A in stretch-induction of mitochondrial complex IV (COX4), and previous studies showing that HIF1A regulates cytochrome oxidase subunits to optimize efficiency of respiration in hypoxic cells [60], we next performed an analysis of COX4 activity along with the protein expression of the cytochrome oxidase subunit COX4-2 during ALI. Here, we found a very robust increase of COX4 activity and the induction of COX4-2 protein levels in control mice exposed to ALI (Figure 10C,D). In contrast, in vivo studies using *Hif1a^f/f^* SurfactantCre+ mice during ALI demonstrated that the observed induction was completely abolished in mice with alveolar epithelial deletion of HIF1A. Accordingly, increases in pulmonary ATP levels following ALI were abolished in *Hif1a^f/f^* SurfactantCre+ mice (Figure 10E). Consistent with a mitochondrial dysfunction in *Hif1a^f/f^* SurfactantCre+ mice during ALI, we found higher ROS levels (Figure 10F). Similarly, complete inhibition of mitochondrial respiration utilizing rotenone treatment in wild-type animals exposed to ventilator associated lung injury was associated with dramatically increased lung inflammation (IL-6 levels displayed; Figure 10G) or alveolar lung leakage (BAL albumin; Figure 10H), thereby mimicking our previous findings in *Hif1a^f/f^* SurfactantCre+ mice. Taken together, these studies indicate that HIF-dependent optimization of glucose uptake, glycolysis, TCA flux, and respiration of the alveolar epithelium represents an endogenous adaptive process that counter-regulates pathologic lung inflammation and improves survival during ventilator-induced ALI (Figure 11A).

## Discussion

The present study was designed to identify stress-elicited transcriptional pathways that could be targeted as novel treatment for ALI. For this purpose, we performed a genome-wide screen to identify transcriptional responses to cyclic mechanical stretch of pulmonary epithelial cells, such as occurs during mechanical ventilation in the context of ALI. Quite surprising, these studies revealed a transcriptional response that resembled hypoxia signaling. We found that the key transcription factor for mediating hypoxia adaptation—HIF1A—was stabilized during stretch conditions in vitro, or during ALI induced by mechanical ventilation in vivo. Unexpectedly, HIF1A stabilization occurred in the absence of tissue hypoxia, and involved normoxic inhibition of SDH, a molecular pathway that has previously been implicated in the normoxic activation of HIF during cancer [46]–[48]. In order to define the functional role of HIF1A during ALI, we subsequently performed pharmacologic studies with HIF activators or inhibitors. These findings demonstrated a protective role for HIF stabilization during ALI. Systematic deletion of the *Hif1a* gene in different lung tissues identified a predominant role for alveolar epithelial HIF1A signaling in these responses. Subsequent metabolic studies indicated that stretch exposure of pulmonary epithelial cells in vitro or ventilator-induced ALI in vivo is associated with the normoxic stabilization of HIF1A, thereby leading to increased glycolytic capacity, TCA flux, optimized mitochondrial respiration, and finally increased ATP generation. Indeed, HIF-dependent prevention of mitochondrial dysfunction during ALI resulted in increased alveolar epithelial capacity to produce ATP, while concomitantly preventing ROS accumulation and lung inflammation (Figure 11).

We were surprised to find a functional role for pulmonary hypoxia-signaling during ALI—particularly because the lungs represent one of the best oxygenated environments in the body. However, this is not the first time that studies uncovered a surprising manifestation for hypoxia-signaling under normoxic conditions. As such, lactate and pyruvate have been shown to regulate hypoxia-inducible gene expression independently of hypoxia by stimulating the accumulation of HIF1A in human gliomas and other cancer cell lines [75]. In contrast to those studies, we observed that HIF stabilization occurred during ALI under normoxic conditions. Combinations of in vitro stretch studies of pulmonary epithelial cells and in vivo studies of ALI induced by mechanical ventilation implicate stretch-elicited inhibition of SDH in HIF activation. These findings are consistent with previous studies of SDH-dependent HIF activation [46]–[48]. Studies on mutations in human cancers had implicated SDH in altered cellular metabolism and cellular transformation. Inactivating SDH causes the accumulation of succinate, which inhibits 2-oxoglutarate-dependent enzymes, including PHDs that mark HIF1A for polyubiquitylation and proteasomal degradation [48]. In support of these findings, other studies have redundantly shown the critical role of metabolism in regulating HIF1A-dependent gene expression [76].

Also consistent with the current studies, previous studies have shown molecular pathways for normoxic stabilization of HIF1A. For example, a recent study described hypoxia-independent activation of HIF1A by enterobacteriaceae and their siderophores [77]. Several studies had provided evidence for HIF1A stabilization during bacterial infections [78]–[80]. In extension of these findings, the authors pursued the role of bacterial siderophores in HIF1A activation during infection with Enterobacteriaceae. Infection of mice with *Y. enterocolitica* led to functional activation of HIF1A in Peyer's patches. Because mice with deletion of HIF1A in the intestinal epithelium showed a significantly higher susceptibility to orogastric *Y. enterocolitica* infections, activation of HIF1A in host cells during bacterial infection represents a host defense mechanism in this study. Additional studies with *Y. enterocolitica*, *S. enterica subsp enterica*, or *E. aerogenes*, and moreover, application of their siderophores (yersiniabactin, salmochelin, aerobactin), caused a robust, dose-dependent HIF1A response in human epithelia and endothelia, independent of cellular hypoxia [77]. The authors conclude that bacterial siderophores account for the normoxic stabilization of HIF1A during infection with human pathogenic bacteria.

In the context of lung injury, previous studies had shown an indirect role for hypoxia-signaling in attenuating ALI induced by bacterial infection [81]. For example, an important study tested the hypothesis that oxygenation weakens a tissue-protecting mechanism triggered by hypoxia and concomitant HIF activation [41]. The authors found that the hypoxia-dependent transcriptional control of extracellular adenosine signaling protects the lungs from the toxic effects of overactive immune cells such as neutrophils [35]. In line with the present studies, these findings demonstrate a protective role for the activation of the HIF-pathway in the lungs by attenuating pathologic lung inflammation during ALI. These studies pointed towards a protective role for HIF-elicited increases of adenosine signaling through the Adora2a adenosine receptor [7],[8]. During conditions of ischemia or inflammation, different cells release nucleotides that can be enzymatically converted to adenosine [2],[23],[38],[82]–[85]. HIF1A can function in multiple ways to enhance the protective effects of extracellular adenosine signaling during ALI, including enhanced enzymatic adenosine production by transcriptionally inducing the ecto-5′-nucleotidase (conversion of extracellular AMP to adenosine), which is a known HIF target gene [86],[87]. Similarly, other studies had shown a protective role for the extracellular production of adenosine and signaling events through adenosine receptors in lung protection—for example, by attenuating vascular leakage [88]–[91] or improving alveolar fluid transport [40]. Moreover, other studies also implicate HIF in attenuating adenosine uptake via repression of equilibrative nucleoside transporters, and thereby enhancing extracellular-dependent tissue protection via adenosine signaling events [92]–[95]. Interestingly, CD73 was among the genes regulated during stretch exposure of pulmonary epithelial cells in our present study (Table S1). Consistent with a role of HIF-dependent increases of CD73-dependent adenosine generation and lung protection during ALI, we also found that the protective effects of the HIF activator DMOG was attenuated in gene-targeted mice for *cd73* (Figure S8). Taken together, these studies indicate the likelihood that HIF-dependent lung protection during ALI may also involve HIF-elicited increases in extracellular adenosine receptor signaling [96],[97]. However, it remains presently unclear if the findings of HIF1A-elicited improvements in carbohydrate metabolism of pulmonary epithelial cells are related or independent of adenosine metabolism and signaling. Moreover, it will be important to examine the role of this pathway in additional models of lung injury, such as ischemia reperfusion and second organ reflow injury of the lung [98] or in models of inflammatory lung disease [99],[100].

Other previous studies had implicated a functional role of HIF-dependent induction of glycolysis in inflammatory diseases. For example, previous studies had shown that HIF signaling is required for myeloid cell metabolism, and for their capability to function during conditions of inflammatory hypoxia [70]. These studies demonstrated that stabilization of HIF1A is essential for myeloid cell infiltration and activation in vivo via functioning as a essential regulator for their glycolytic capacity: when HIF1A is absent, the metabolic defect results in profound impairment of myeloid cell aggregation, motility, invasiveness, and bacterial killing [70]. However, during conditions of ALI—such as those that were used in the present studies—the lungs remain normoxic, and PMN-dependent HIF stabilization and HIF-elicited increases of PMN-inflammatory functions appear to be an unlikely scenario. In contrast, stretch-induced stabilization of HIF1A under normoxic conditions in epithelial cells appears to contribute to lung protection during ALI. Indeed, HIF-dependent increases in alveolar epithelial cell glycolysis, TCA cycle flux, and mitochondrial respiration allows for a metabolic adaptation that is critical to dampen pathologic lung inflammation during ventilator-induced lung injury.

Consistent with the present studies implicating HIF in mitochondrial respiration during ALI, a previous study had demonstrated a functional role of HIF1A in mitochondrial respiration via altering mitochondrial complex IV (COX4) subunit composition under hypoxic conditions [60]. Moreover, the current metabolic in vivo studies in mice with tissue-specific deletion of HIF1A in alveolar epithelia are consistent with previous studies from the 1970s that examined normoxic or hypoxic metabolism of cultured pulmonary epithelia. During air cultivation, alveolar epithelial cells had a high rate of aerobic and anaerobic glycolysis [101]. However, the authors observed concomitant increases of anaerobic and aerobic glycolysis under hypoxic condition [101]. These findings are consistent with the present studies showing that HIF1A is responsible for increases of glycolysis and lactate production and, at the same time, for oxidative glucose flux rates during ALI in vivo. Our findings utilizing liquid chromatography–tandem mass spectrometry analysis of metabolites following the infusion of ^13^C-glucose-labeled glucose in mice with tissue-specific deletion of alveolar epithelial HIF1A link these early observations with a functional role for HIF1A in controlling alveolar epithelial metabolism during ALI.

Taken together, the present studies demonstrate a critical role for the stabilization of HIF1A during conditions of cyclic mechanical stretch in vitro, or during ALI in vivo, representing an endogenous protective mechanism. We conclude that targeting HIF-dependent metabolism of alveolar epithelia during ALI represents a powerful therapeutic strategy to dampen lung inflammation. These findings are also interesting from a translational perspective: a pharmacologic activator of HIF has recently been studied in the treatment of patients with renal anemia, and there were no safety concerns observed [49]. As such, treatment of ALI with HIF activators could potentially be considered in a clinical trial of ALI. Based on the specificity of our findings for pulmonary epithelial HIF1A, an inhaled treatment approach with less systemic concentrations of the compound may also be feasible.

## Materials and Methods

### Ethics Statement

Experimental protocols were approved by the Institutional Review Board at the University of Colorado Anschutz Medical Campus, Aurora, Colorado. They were in accordance with the U.S. Law on the Protection of Animals and the National Institutes of Health guidelines for use of live animals.

### Cell Culture and Treatments

Calu-3 human airway epithelial cells or human pulmonary epithelial A549 cells were cultured as described previously [23],[40]. Primary pulmonary epithelial cells (HPAEpiC) (ScienCell Research Laboratories, California) were cultured according to the supplier's instructions.

### In Vitro Stretch Model

To study the consequences of cyclic mechanical stretch, we adopted a previously described in vitro model resembling mechanical ventilation by applying cyclic mechanical stretch. In short, Calu-3, A549, or HPAEpiC were plated on BioFlex culture plates-collagen type I (BF-3001C; FlexCell International) and allowed to attach and grow to 80% confluence. All cells were cultured in 4 ml media: Calu-3 cells were grown in Advanced MEM (GIBCO), A549 cells were grown in DMEM-F12 (GIBCO), both cell lines with 10% FBS, 0.02% L-Glutamine. Plates were then placed on a FlexCell FX-4000T Tension Plus System and stretched at percentage stretch indicated, 30% maximum, 0.7% stretch minimum, and sine wave 5 s on, 5 s off. Cells were collected at indicated time points from duplicate wells, flash-frozen, and stored at −80°C for further analysis. For control, cells were cultured under similar conditions at rest (no cyclic mechanical stretch).

### Microarray Analysis

Array data have been deposited at GEO (accession number GSE 27128) (http://www.ncbi.nlm.nih.gov/projects/geo/query/acc.cgi?acc=GSE27128). The transcriptional profile in Calu-3 cells subjected to 30% stretch for 24 h was assessed from total RNA (isolated using Qiagen RNeasy kit) using quantitative GeneChip arrays (Human genome U133 Plus 2.0 Array). The integrity of RNA was assessed using an Agilent 2100 Bioanalyzer (Agilent Technologies), and RNA concentration was determined using a NanoDrop ND-1000 spectrophotometer (NanoDrop, Rockland, Delaware). Biotinylated cRNAs for hybridization to Affymetrix 3′-arrays were prepared from total RNA using the Affymetrix two-cycle target labeling assay with spike in controls (Affymetrix Inc., Santa Clara, California). Labeled-cRNA was fragmented and hybridized to Human Genome Arrays following the manufacturer's protocols. After hybridization and staining, the arrays were scanned using a GCS3000 with the latest 7G upgrade. Each array was subjected to visual inspection for gross abnormalities. Several other QC metrics were used to monitor hybridization efficiency and RNA integrity over the entire processing procedure. Raw image files were processed using Affymetrix GCOS 1.3 software to calculate individual probe cell intensity data and generate CEL data files. Using GCOS and the MAS 5.0 algorithm, intensity data were normalized per chip to a target intensity TGT value of 500 and expression data and present/absent calls for individual probe sets calculated. Quality control was performed by examining raw DAT image files for anomalies, confirming each GeneChip array had a background value of less than 100, monitoring that the percentage present calls was appropriate for the cell type, and inspecting the poly(A) spike in controls, housekeeping genes, and hybridization controls to confirm labeling and hybridization consistency. According to our experimental setup, the arrays were normalized, grouped, and analyzed for differentially expressed transcripts based on different statistical tests. Different clustering algorithms allowed us to identify transcripts that show similar expression profiles. Using the “Ingenuity Pathway Analysis,” we were able to identify biological mechanisms, pathways, and functions most relevant to our experimental dataset (Table S1, Figure S1).

### Immunoblotting and Immunohistochemistry Experiments

All antibodies used were anti-HIF1A mouse monoclonal [H1alpha67] (Abcam), anti-HIF1A mouse monoclonal [H1alpha67] (Novus), anti-HIF2A rabbit polyclonal (Novus), and anti-COX 4|2 mouse monoclonal (R&D). COX4|2 immunoblotting was performed using mitochondrial protein fractions.

### Lentiviral-Mediated Generation of Cells with Knockdown of HIF1A and HIF2A

Stable cell cultures with decreased HIF1A and HIF2A expression were generated by lentiviral-mediated shRNA expression. pLKO.1 lentiviral vectors targeting HIF1A had shRNA sequence of CCG GCC AGT TAT GAT TGT GAA GTT ACT CGA GTA ACT TCA CAA TCA TAA CTG GTT TTT (TRCN 0000003809), and HIF2A had a sequence CCG GCC ATG AGG AGA TTC GTG AGA ACT CGA GTT CTC ACG AAT CTC CTC ATG GTT TTT (TRCN 0000003807). For controls, nontargeting control shRNA (SHC002;Sigma) was used. HEK293T cells were co-transfected with pLK0.1 vectors and packaging plasmids to produce lentivirus. Filtered supernatants were used for infection of Calu-3 and cells were selected with puromycin (30 ug ml^−1^) for at least two passages before initiating stretch experiments.

### Transcriptional Analysis

Total RNA was isolated from human lung epithelia or murine lung tissue and transcript levels were determined by real-time RT-PCR (iCycler; Bio-Rad Laboratories Inc.) [90]. Primers were Quantitect from Qiagen.

### Lactate Measurements and Blood Gas Analysis

To assess partial oxygen pressures (pO_2_) and lactate from cell supernatants or to determine pulmonary gas exchange (paO_2_) in mice from arterial blood obtained via cardiac puncture, samples were analyzed immediately after collection with the I-STAT Analyzer (Abbott).

### NMR Analysis on Stretched Calu-3 Cells

All 1H-NMR spectra were obtained at the Bruker 500 MHz DRX NMR spectrometer using an inverse Bruker 5-mm TXI probe. High-resolution ^1^H- and ^13^C-NMR experiments were performed with the Bruker 500 MHz DRX spectrometer equipped with an inverse 5-mm TXI probe (Bruker BioSpin, Fremont, California) and ^31^P-NMR experiments with the 300 MHz Bruker Avance system with a 5-mm QNP probe. For proton NMR, a standard water presaturation pulse program was used for water suppression; spectra were obtained at 12 ppm spectral width, 32K data arrays, 64 scans with 90-degree pulses applied every 12.8 s. Trimethylsilyl propionic-2,2,3,3,-d4 acid (TSP, 0.5 mmol/L) was used as an external standard for metabolite chemical shift assignment (0 ppm) and quantification. ^13^C-NMR spectra with proton decoupling were recorded using the C3-lactate peak at 21 ppm as chemical shift reference (spectral width was 150 ppm, 16K data arrays, 20K scans applied every 3 s). [3-^13^C] lactate satellite peak (at 1.23 ppm) from 1H-NMR spectra served as an internal standard for ^13^C-NMR spectra (at 21 ppm) for calculation of ^13^C-enrichment of glucose and glucose metabolites. ^31^P-NMR spectra were obtained using the spectral width of 50 ppm and 16K data arrays, with 6K–10K scans being applied every 3.5 s. Before ^31^P-NMR spectra were recorded, EDTA (100 mmol/L) was added to each PCA extract to complex divalent cations. Methylene diphosphonic acid (2 mmol/L) was used as an external standard for chemical shift references (18.6 ppm) and for metabolite quantification in ^31^P-NMR. For nuclear magnetic resonance (NMR) analysis of metabolites, cells were shock frozen immediately after stretch. Rate of glycolysis or TCA cycle flux is given as ratio of incorporated ^13^C intermediates of glycolysis compared to ^13^C glucose levels. All data were processed using the Bruker WINNMR program. All NMR experiments were performed at the Metabolomics NMR University of Colorado Cancer Center Core.

### Mitochondrial Complex II and IV and PDH Activity Measurement

Succinate Dehydrogenase (SDH, Complex II Enzyme Activity Microplate Assay Kit, Abcam) or COX4 (Complex IV Enzyme Activity Microplate Assay Kit, Abcam) or Pyruvate Dehydrogenase (Pyruvate dehydrogenase (PDH) Enzyme Activity Microplate Assay Kit) activities were determined from mitochondrial extracts using enzyme activity assays manufactured by Abcam.

### SUCLG Suppression with RNA Interference

A549 cells were either grown on inserts or in 60-mm Petri dishes. SMARTpool siRNA targeting SUCLG was synthesized by Dharmacon (Lafayette, Colorado). SMART pool reagents combine four SMART selection-designed siRNAs into a single pool, resulting in even greater probability that the SMARTpool reagent will reduce target mRNA to low levels. In addition, to further increase transfection efficiency and off-target effects, siRNA was selected by Dharmacon according ON-TARGETplus criteria. As control, siRNA (Dharmacon, Lafayette, Colorado) with at least four mismatches to any human, mouse, or rat gene was used. A549 cell loading was accomplished using standard conditions of DharmaFECT (Dharmacon, Lafayette, Colorado), when cells had reached 40–60% confluence. After 48 h of loading, RNA/Protein was isolated as described before [102].

### Succinate-Mediated PHD Inhibition

Octyl-α-ketoglutarate as a stable, cell-permeable form of α-ketoglutarate that accumulates rapidly and preferentially in cells with a dysfunctional TCA cycle was used at 1 mM.

### Pharmacological Inhibition of MAP Kinases

To inhibit MAP kinases *in vitro*, AMG 548 [Tocris, potent and selective inhibitor of p38 (at 10 µM inhibits p38α, p38β, p38γ, and p38δ)], FR 180204 [Tocris, selective ERK inhibitor (at 1 µM inhibits ERK2 and ERK1)], and BI 78D3 [Tocris, competitive c-Jun N-terminal kinase (JNK) inhibitor (500 nM)] was used.

### TNFA and IL6 Neutralizing

For TNF-α neutralizing, D1B4 (Cell Signaling) Rabbit mAb was used. For IL-6, anti-IL-6 antibody (ab6672) from abcam was used.

### ELISA (IL-6, IL-8, KC, TNF-α, MPO) from Cells, Lung Tissue, or BAL

The snap-frozen lungs were thawed, weighed, and transferred to different tubes on ice containing 1 ml of Tissue Protein Extraction Reagent (T-PER; Pierce Biotechnology). Cells or lung tissues were homogenized at 4°C. Cell or lung homogenates were centrifuged at 9,000 g for 10 min at 4°C. Supernatants were transferred to clean microcentrifuge tubes, frozen on dry ice, and thawed on ice. Total protein concentrations in the lung tissue homogenates were determined using a bicinchoninic acid kit (Pierce Biotechnology). IL-6 (R&D Systems), IL-8 (R&D Systems), KC (R&D Systems), TNF-α (R&D Systems), and MPO (Hycult Biotech) levels were evaluated in lung tissue homogenates using a mouse ELISA kit according to the user's manual.

### ATP Measurements

ATP from cells or tissue was determined using ATP Bioluminescence Assay Kit CLS II for ATP Measurement from Roche.

### ROS Measurements

Intracellular hydrogen peroxide levels were measured using an Amplex Red Hydrogen Peroxide Assay kit (Molecular Probes/Invitrogen) according to the manufacturer's instructions. In brief, total cell lysates and supernatants were harvested 24 h after stretch in the FlexCell FX-4000T Tension Plus System. Lung tissue exposed to VILI was homogenized in Tissue Protein Extraction Reagent (T-PER; Pierce Biotechnology) and spun down at 13,000 g at 4°C for 10 min. Supernatants were collected and used in the assay. Bronchoalveolar lavages from VILI exposed animals were collected in 1 mL of saline. On a 96-well microplate, samples were added and reactions were initiated immediately by adding Amplex Red reaction mixture. Fluorescence was measured on Synergy 2 Multi-Mode Microplate reader (BioTek) in excitation range of 540/25 and emission detection of 620/40. Fluorescence levels were normalized to the protein concentration.

### Mice

Wild-type mice (BL6C57), Hif1a reporter mice (FVB.129S6-*Gt(ROSA)26Sor^tm2(HIF1A/luc)Kael^*/J) [63], *Hif1a^f/f^(B6.129-Hif1atm3Rsjo/J)*, ActinCre+ (B6.Cg-Tg(CAG-cre/Esr1*)5Amc/J) [103], CadherinCre+ (B6.Cg-Tg(Cdh5-cre)7Mlia/J) [104], SPC-rtA (B6.Cg-Tg(SFTPC-rtTA)5Jaw/J) [105], and Tet-O-Cre (B6.Cg-Tg(tetO-cre)1Jaw/J) [106] were purchased from Jackson laboratories. Cre exclusively expressed in the conducting airway (ClaraCellCre+) were obtained from Thomas Mariani [107]. To obtain specific *Hif1a* ^−/−^ mice, *Hif1a^f/f^* mice were crossed with the appropriate Cre mouse. Whole body *Hif1a* ^−/−^ mice knockout were achieved by a 5-d treatment of tamoxifen (1 mg/d) i.p. For Hif1a tissue-specific knockout in the alveolar epithelium, triple transgenic mice (*Hif1a^f/f^* SPC-rtA Tet-O-Cre) were induced by Doxycyclin therapy over 5 d i.p and p.o. as described [106].

### Murine Mechanical Ventilation

All animal protocols were in accordance with the guidelines of the National Institute for Health for the use of laboratory animals and approved by the Institutional Animal Care and Use Committee of the University of Colorado. Ventilator-induced lung injury was induced as described previously [40].

### BAL Lavage

To obtain BAL fluid, the tracheal tube was disconnected from the mechanical ventilator and the lungs were lavaged 3 times with 0.5 ml of PBS. All removed fluid was centrifuged immediately, and the supernatant was aliquoted for measurement of the albumin concentration.

### Detecting Luciferase Expression in Vivo

Mice were given a single i.p. injection of a mixture of luciferin (50 mg/kg) in sterile water. Following normal ventilation (15 mbar, 3 h) or VILI (45 mbar, 3 h), lungs were immediately excised and placed in a light-tight chamber equipped with a charge-coupled device IVIS imaging camera (Xenogen, Alameda, California). Photons were collected for a period of 5–20 s, and images were obtained by using living image software (Xenogen) and igor image analysis software (WaveMatrics, Lake Oswego, Oregon).

### Luciferase Assay—Tissue

Expression of the Hif1a reporter genes was assayed using the tissue homogenates in Tper Tissue Protein Extraction Reagent (Pierce). The homogenates were centrifuged for 30 min at 4,900×g at 4°C. The luciferase gene expression was measured by using the Dual-Luciferase Reporter Assay System from Promega according to the manufacturer's instructions using a Biotek Synergy 2 Multimode Microplate Reader.

### Pharmacolgical Studies in Vitro and in Vivo

Rotenone (Tocris), 2-Deoxy-D-glucose (2-DG), Dimethyloxalylglycine (DMOG), and Echinomycin were purchased from Sigma-Aldrich, USA and used at the concentrations as indicated.

### Isolation of Murine Alveolar Epithelial Cells

Following 4 h of VILI, mice were euthanized by pentobarbital overdose. Lungs were lavaged with sterile PBS, then perfused with PBS via the right ventricle. As previously described, dispase (BD, USA) was instilled followed by a low-melting point agarose plug. Lungs were removed intact and incubated at 37°C for 30 min. Tissue was dissociated manually and cells progressively filtered (70 µm/40 µm, Fisher, USA). Cells were treated with FcR blocking mAB (24G2) and labeled with anti-EpCAM biotin (eBioscience, USA) and anti-biotin Dylight 633. EpCAM positive cells were positively selected using streptavidin magnetic microbeads (Miltenyi, USA). Purified cells were analyzed for EpCAM expression using a LSRII flow cytometer (BD, USA) and FlowJo v8.8.4 software (TreeStar, USA) [108].

### Histopathological Evaluation of ALI

Following ventilation at the settings indicated in the figure legends, the mice were euthanized and lungs were fixed by instillation of 10% formaldehyde solution via the tracheal cannula at a pressure of 20 mbar. Lungs were then embedded in paraffin and stained with hematoxylin and eosin. Two random tissue sections from four different lungs in each group were examined by a pathologist who was blinded to the genetic background and treatment of the mice. For each subject, a 5-point scale was applied: 0, minimal damage; 1 to >2, mild damage; 2 to >3, moderate damage; 3 to >4, severe damage; and 4+, maximal damage. Points were added together and are expressed as median ± range (*n* = 4).

### Metabolite Analysis by UPLC-MS

*Hif1a^f/f^* SPC-rtA Tet-O-Cre mice or littermate controls (induced SPC-rtA Tet-O-Cre mice) matched in age, weight, and gender were exposed to 2 h of VILI [45 mbar]. ^13^C-glucose was administered 30 min prior to the onset of VILI via intraperitoneal injection. Lung tissue was snap-frozen with clamps pre-cooled to the temperature of liquid nitrogen. Seven metabolites (glucose, lactate, pyruvate, malate, fructose-1,6-bisphosphate, glutamate, and creatine) were measured by ultrahigh performance liquid chromatography–mass spectrometry (UPLC-MS) using a Waters Acquity ultrahigh-performance liquid chromatograph coupled to a Waters Synapt HDMS quadrupole time-of-flight mass spectrometer, which was operated with an atmospheric pressure electrospray ionization (ESI) source as described previously.[32]

### Data Analysis

Data were compared by two-factor ANOVA with Bonferroni's posttest or by Student's *t* test where appropriate. Values are expressed as mean ± s.d. from 3–6 animals per condition. For analysis of changes in transcript, a one-way ANOVA was carried out and multiple comparisons between control and treatment groups were made using the Dunnett posttest. The Mantel Cox test was used for analysis of survival curves. Data are expressed as mean ± s.d.; *p*<0.05 was considered statistically significant. For all statistical analyses, GraphPad Prism 5.0 software for Windows XP was used. The authors had full access to and take full responsibility for the integrity of the data. All authors have read and agree to the manuscript as written.

## Supporting Information

Figure S1 **Pathway analysis.** Human alveolar epithelial cells (Calu-3) were exposed to stretch conditions as an in vitro model for ventilator-induced ALI (24 h stretch at 30% intensity (http://www.ncbi.nlm.nih.gov/projects/geo/query/acc.cgi?acc=GSE27128). Computerized pathway analyses to examine alterations in gene transcription (Ingenuity IPA, Version 11631407) shows that hypoxia-signaling resembled the dominant stress response pathway when comparing stretch-exposed pulmonary epithelia to un-stretched controls.
(TIFF)

Figure S2 **In vitro studies on stretch-induced HIF1A.** (A–C) Stable cell cultures with decreased HIF1A or HIF2A expression were generated by lentiviral-mediated shRNA expression. For controls, nontargeting control shRNA was used. Cells were co-transfected with pLK0.1 vectors and packaging plasmids to produce lentivirus. Filtered supernatants were used for infection of Calu-3 and cells were selected with puromycin (30 mg/ml) for at least two passages before initiating stretch experiments. RT PCR or Western blot for HIF1A or HIF2A revealed a 98% reduction of transcript or protein, respectively.
(TIFF)

Figure S3 **In vitro studies in HIF1A or HIF2A knockdown Calu-3 cells.** (A) Transcript levels of glycolytic enzymes from HIF1A KD Calu-3 pulmonary epithelia (lentivirus-mediated HIF1A knockdown) or Calu-3 epithelia transduced with a control virus (lentiviral scrambled siRNA, Scr) after 24 h of stretch were determined by real-time RT-PCR relative to housekeeping gene beta-actin (mean ± s.d., *n* = 3). (B–D) Calu-3 controls or Calu-3 HIF2KD were exposed to 24 h of stretch. (B) Levels of glycolytic enzymes after 24 h of stretch were determined by real-time RT-PCR relative to Actb (mean ± SD, *n* = 3). (C) Lactate levels in supernatants obtained from Calu-3 with a lentiviral-mediated HIF2A KD after 24 h of stretch. Controls consisted of Calu-3 cells treated with lentiviral scrambled siRNA exposed to the same experimental conditions. (D) IL-6 and IL-8 levels were evaluated in supernatants from stretched Calu-3 controls or Calu-3 HIF2KD exposed to 24 h of stretch using a human enzyme-linked immunosorbent assay (ELISA). Results are presented as mean ± s.d. (*n* = 4, unless stated otherwise).
(TIFF)

Figure S4 **In vivo studies on pharmacological or genetic HIF1A inhibition.** (A) Mice were treated with 1 mg of DMOG i.p. and lungs were harvested after indicated time periods. Resultant Western blots were probed with anti-Hif1a antibody. To control for loading conditions, blots were stripped and re-probed for actin expression. A representative experiment of three is shown. (B) WT mice were exposed to 3 h of VILI with and without Echinomycin pretreatment. Echinomycin blocks the transcriptional binding site of Hif1a. Levels of glycolytic enzymes were determined by real-time RT-PCR relative to Actb and expressed as fold induction relative to sham-operated controls (mean ± SD, *n* = 3). (C) DMOG treatment (1 mg i.p.) in mice with induced deletion of Hif1a in all tissues, including the lungs (Hif1af/f ActinCre+, whole body knockout mice). Lungs were harvested at indicated time points and Western blots were probed with anti-Hif1a antibody. A representative experiment of three is shown.
(TIFF)

Figure S5 **HIF1A immunohistochemistry.** Hif1a immunohistochemistry is shown for untreated wild-type (WT, left side) compared to cell-type-specific Hif1a knock-out tissue (right side) including (A) lung endothelium, (B) bronchial epithelium, and (C) alveolar cells. (A) Untreated WT lung endothelium (left) display several nuclei with considerable HIF1a immunopositivity (black arrows). Even after DMOG treatment, lung endothelial cells (black arrows) of *Hif1a^f/f^*CadherinCre+ animals (right) showed no Hif1a expression confirming the good quality of the knock-out. (B and C) Similar findings were obtained for bronchial epithelia (B, black arrows) and alveolar cells (C, black arrows) showing moderate Hif1a expression in WT animals (left) but absence in corresponding cell-type-specific Hif1a knock-out mice after DMOG treatment. (A–C) Inflammatory cells in cell-type-specific Hif1a knock-out animals, infiltrating inflammatory cells (red arrows) served as internal positive control still exhibiting Hif1a expression. (Original magnification 40× for all images.)
(TIFF)

Figure S6 **Purified alveolar epithelial cells show reduced HIF1A expression in conditional knockout following VILI.** Conditional knockout mice (experimental/E) have a loxP-flanked *Hif1a*, a SPC-*rtTA*, and TetO-*Cre*-recombinase transgene. Littermate controls (control/C) lack the tetO-*Cre* transgene. (A) Purification of alveolar epithelia cells (AECs) from control and experimental animals: cells are highly enriched for expression of EpCAM (epithelial cell adhesion molecule) indicating purity of AECs. (B) Purified AECs show *Cre*-recombinase activity and absence of floxed Hif1a gene (red box) in experimental animals determined by conventional PCR using standard genotyping protocols (Jackson Laboratory, by GeneTyper–Mouse Genotyping Service).
(TIFF)

Figure S7 **Consequences of glycolysis inhibition during ALI.** (A) Transcript levels of phosphofructokinase-m (Pfkm), pyruvate dehydrogenase kinase 1 (Pdk1), and lactate dehydrogenase a (Ldha) from controls (SurfactantCre+) or Hif1af/f SurfactantCre+ mice after 180 min at 45 mbar mechanical ventilation (mean ± s.d., *n* = 3). (B) Hydrogen peroxide levels of BAL from *Hif1a^f/f^* SurfactantCre+ mice or age-, gender-, and weight-matched littermate controls (SurfactantCre+) with or without 2-DG treatment. The data are expressed as the mean fluorescence levels from two independent experiments normalized by protein concentration (ELISA). Results are presented as mean ± s.d. (*n* = 4, unless stated otherwise).
(TIFF)

Figure S8 **Functional consequences of HIF1A activation during ALI in CD73 deficient mice.** (A) HIF1A activator dimethyl-oxaloylglycine (DMOG) during ALI: BL6C57 or CD73−/− mice were treated with 1 mg DMOG or vehicle control 4 h prior to the experimental procedure. Mechanical ventilation was instituted and mice were ventilated for 180 min using pressure-controlled settings (inspiratory pressure of 45 mbar, 100% inspired oxygen concentration). Albumin concentration in the bronchoalveolar fluid (BAL) was determined by enzyme-linked immunosorbent assay. Results are presented as mean ± s.d. (*n* = 6).
(TIFF)

Table S1 **Microarray analysis.** Human alveolar epithelial cells (Calu-3) were exposed to stretch conditions as an in vitro model for ventilator-induced ALI (24 h stretch at 30% intensity; http://www.ncbi.nlm.nih.gov/projects/geo/query/acc.cgi?acc=GSE27128). A subset of HIF1A-dependent genes were confirmed utilizing real-time RT-PCR and Western blotting. Significant changes in gene expression are displayed as fold change (*p*<0.05). Confirmation by real-time RT-PCR and Western blotting (unpublished data).
(XLSX)

## Abbreviations

ALI: acute lung injury
COX4: mitochondrial complex IV
DMOG: dimethyl-oxaloylglycine
HIF1A: hypoxia-inducible factor 1A
LDHA: lactate dehydrogenase a
MAPK: mitogen-activated protein kinase
PDC: pyruvate dehydrogenase complex
PDK1: pyruvate dehydrogenase kinase 1
PFKM: phosphofructokinase m
PHD: prolylhydroxylase
SDH: succinate dehydrogenase
TCA: tricarboxylic acid
