# Supplementary figures and images for: HIF1A Reduces Acute Lung Injury by Optimizing Carbohydrate Metabolism in the Alveolar Epithelium
Source: PLoS Biol. 2013 Sep 24;11(9):e1001665. doi: 10.1371/journal.pbio.1001665 (PMC3782424; doi:10.1371/journal.pbio.1001665)

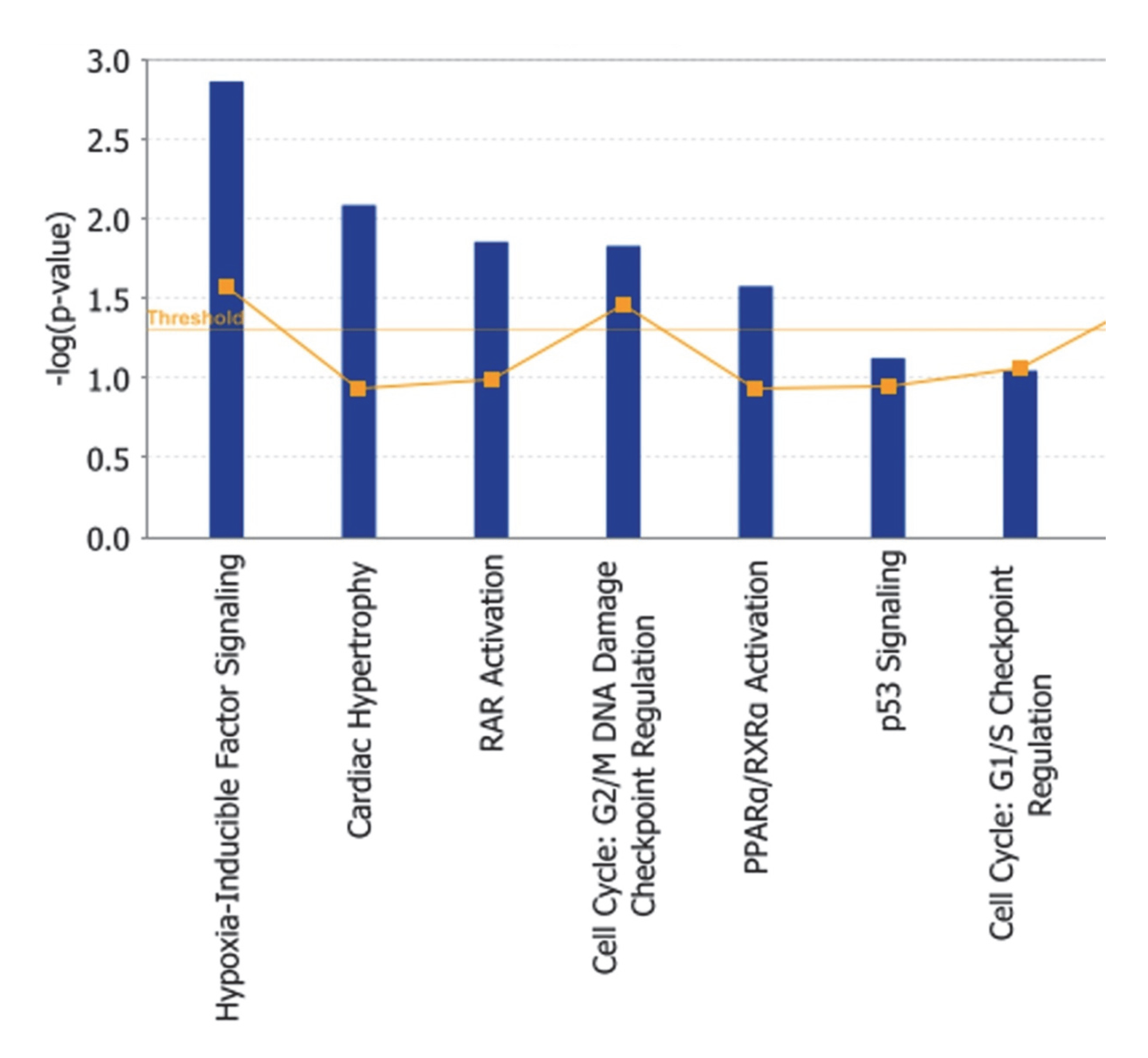

Supplement: Figure S1 — Pathway analysis. Human alveolar epithelial cells (Calu-3) were exposed to stretch conditions as an in vitro model for ventilator-induced ALI (24 h stretch at 30% intensity (http://www.ncbi.nlm.nih.gov/projects/geo/query/acc.cgi?acc=GSE27128). Computerized pathway analyses to examine alterations in gene transcription (Ingenuity IPA, Version 11631407) shows that hypoxia-signaling resembled the dominant stress response pathway when comparing stretch-exposed pulmonary epithelia to un-stretched controls. (TIFF) [file pbio.1001665.s001.tiff]

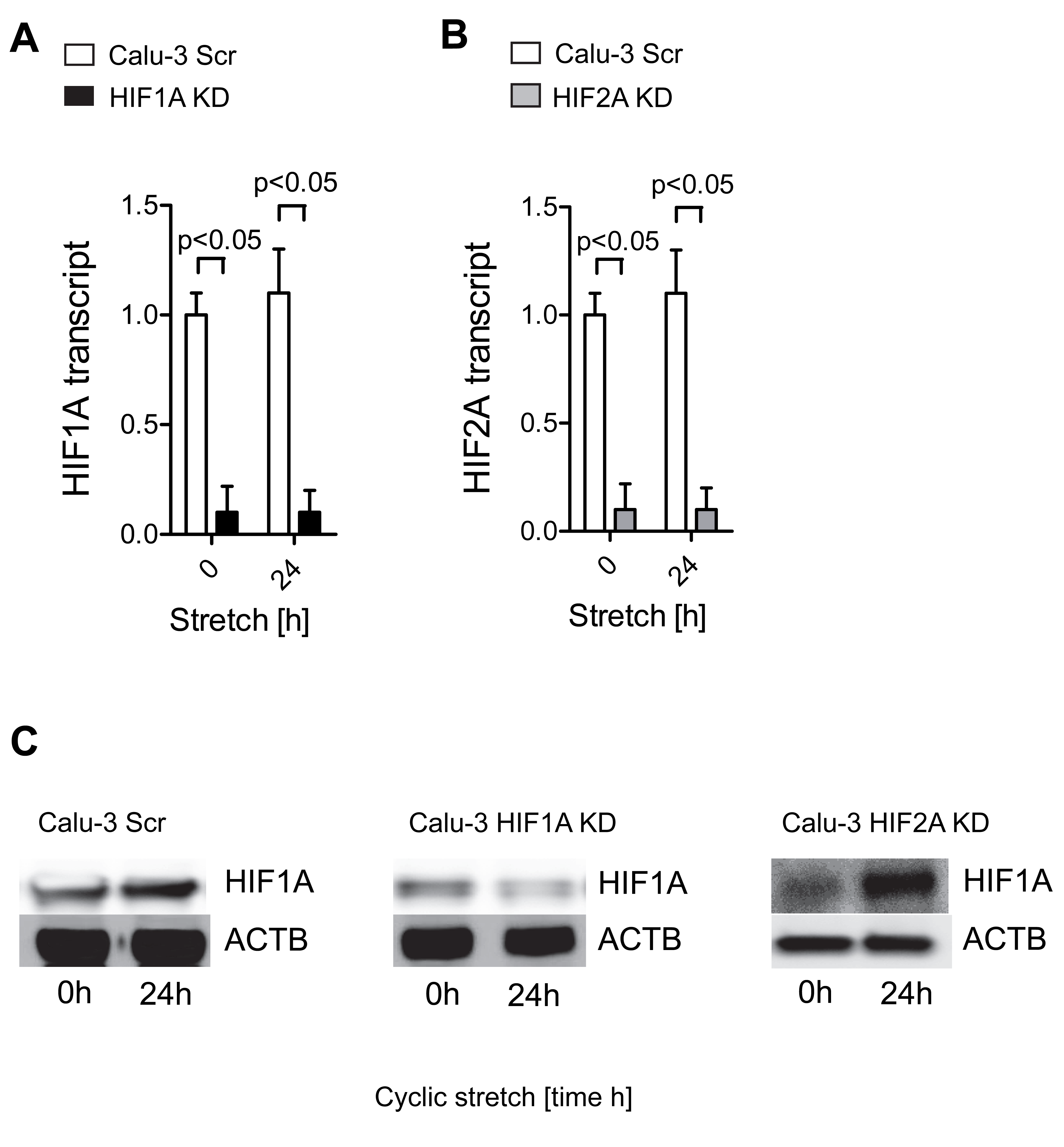

Supplement: Figure S2 — In vitro studies on stretch-induced HIF1A. (A–C) Stable cell cultures with decreased HIF1A or HIF2A expression were generated by lentiviral-mediated shRNA expression. For controls, nontargeting control shRNA was used. Cells were co-transfected with pLK0.1 vectors and packaging plasmids to produce lentivirus. Filtered supernatants were used for infection of Calu-3 and cells were selected with puromycin (30 mg/ml) for at least two passages before initiating stretch experiments. RT PCR or Western blot for HIF1A or HIF2A revealed a 98% reduction of transcript or protein, respectively. (TIFF) [file pbio.1001665.s002.tiff]

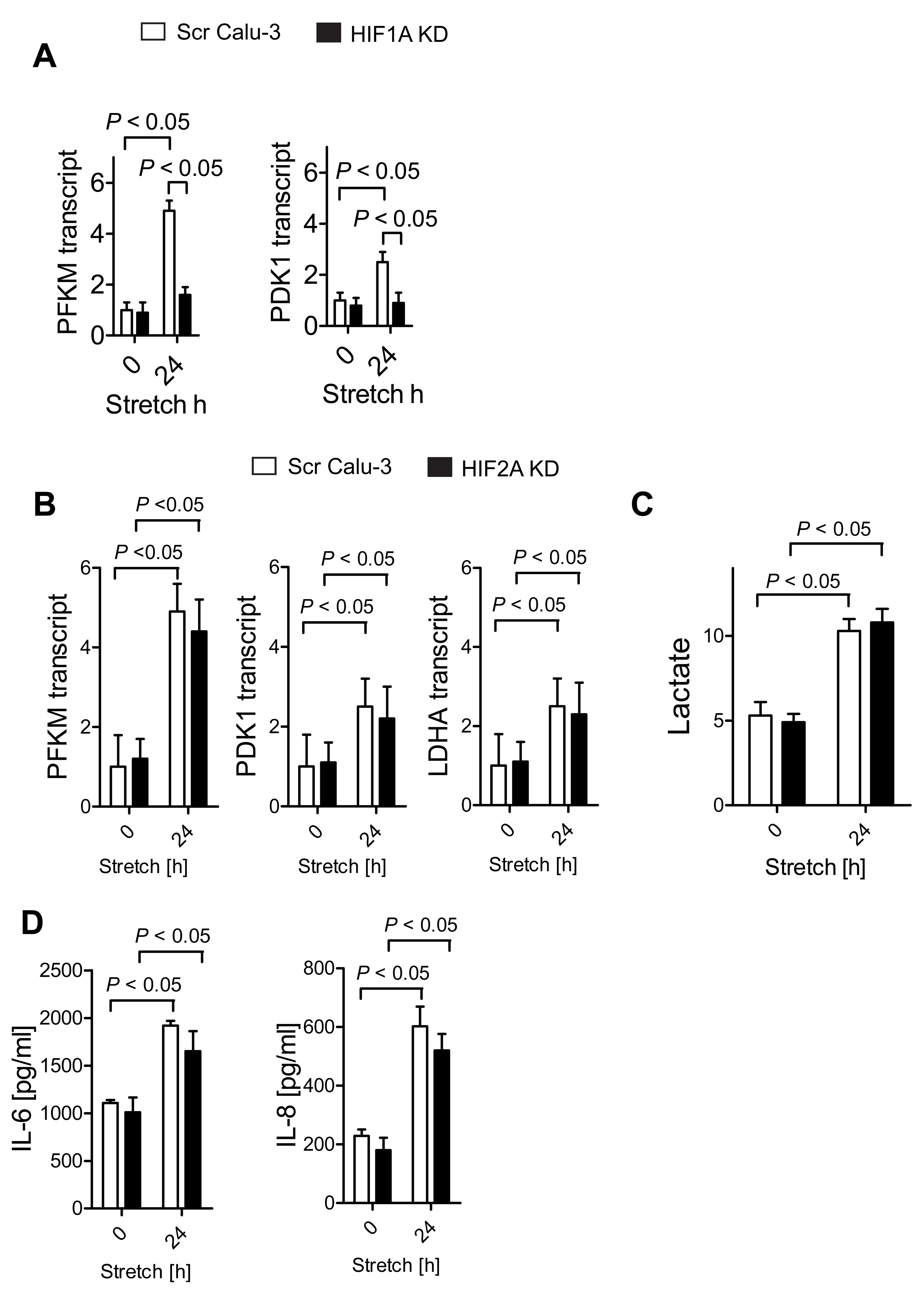

Supplement: Figure S3 — In vitro studies in HIF1A or HIF2A knockdown Calu-3 cells. (A) Transcript levels of glycolytic enzymes from HIF1A KD Calu-3 pulmonary epithelia (lentivirus-mediated HIF1A knockdown) or Calu-3 epithelia transduced with a control virus (lentiviral scrambled siRNA, Scr) after 24 h of stretch were determined by real-time RT-PCR relative to housekeeping gene beta-actin (mean ± s.d., n = 3). (B–D) Calu-3 controls or Calu-3 HIF2KD were exposed to 24 h of stretch. (B) Levels of glycolytic enzymes after 24 h of stretch were determined by real-time RT-PCR relative to Actb (mean ± SD, n = 3). (C) Lactate levels in supernatants obtained from Calu-3 with a lentiviral-mediated HIF2A KD after 24 h of stretch. Controls consisted of Calu-3 cells treated with lentiviral scrambled siRNA exposed to the same experimental conditions. (D) IL-6 and IL-8 levels were evaluated in supernatants from stretched Calu-3 controls or Calu-3 HIF2KD exposed to 24 h of stretch using a human enzyme-linked immunosorbent assay (ELISA). Results are presented as mean ± s.d. (n = 4, unless stated otherwise). (TIFF) [file pbio.1001665.s003.tiff]

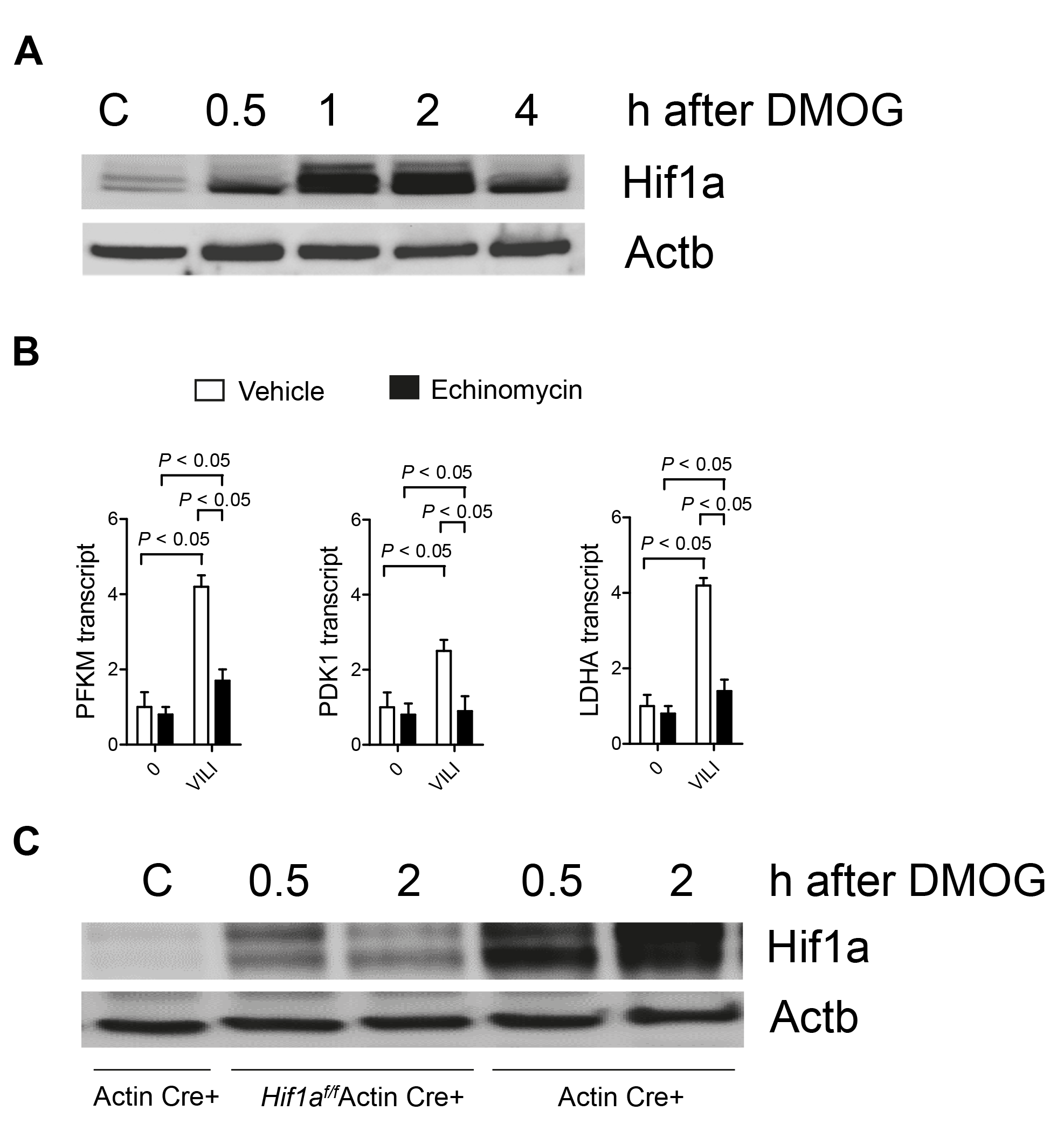

Supplement: Figure S4 — In vivo studies on pharmacological or genetic HIF1A inhibition. (A) Mice were treated with 1 mg of DMOG i.p. and lungs were harvested after indicated time periods. Resultant Western blots were probed with anti-Hif1a antibody. To control for loading conditions, blots were stripped and re-probed for actin expression. A representative experiment of three is shown. (B) WT mice were exposed to 3 h of VILI with and without Echinomycin pretreatment. Echinomycin blocks the transcriptional binding site of Hif1a. Levels of glycolytic enzymes were determined by real-time RT-PCR relative to Actb and expressed as fold induction relative to sham-operated controls (mean ± SD, n = 3). (C) DMOG treatment (1 mg i.p.) in mice with induced deletion of Hif1a in all tissues, including the lungs (Hif1af/f ActinCre+, whole body knockout mice). Lungs were harvested at indicated time points and Western blots were probed with anti-Hif1a antibody. A representative experiment of three is shown. (TIFF) [file pbio.1001665.s004.tiff]

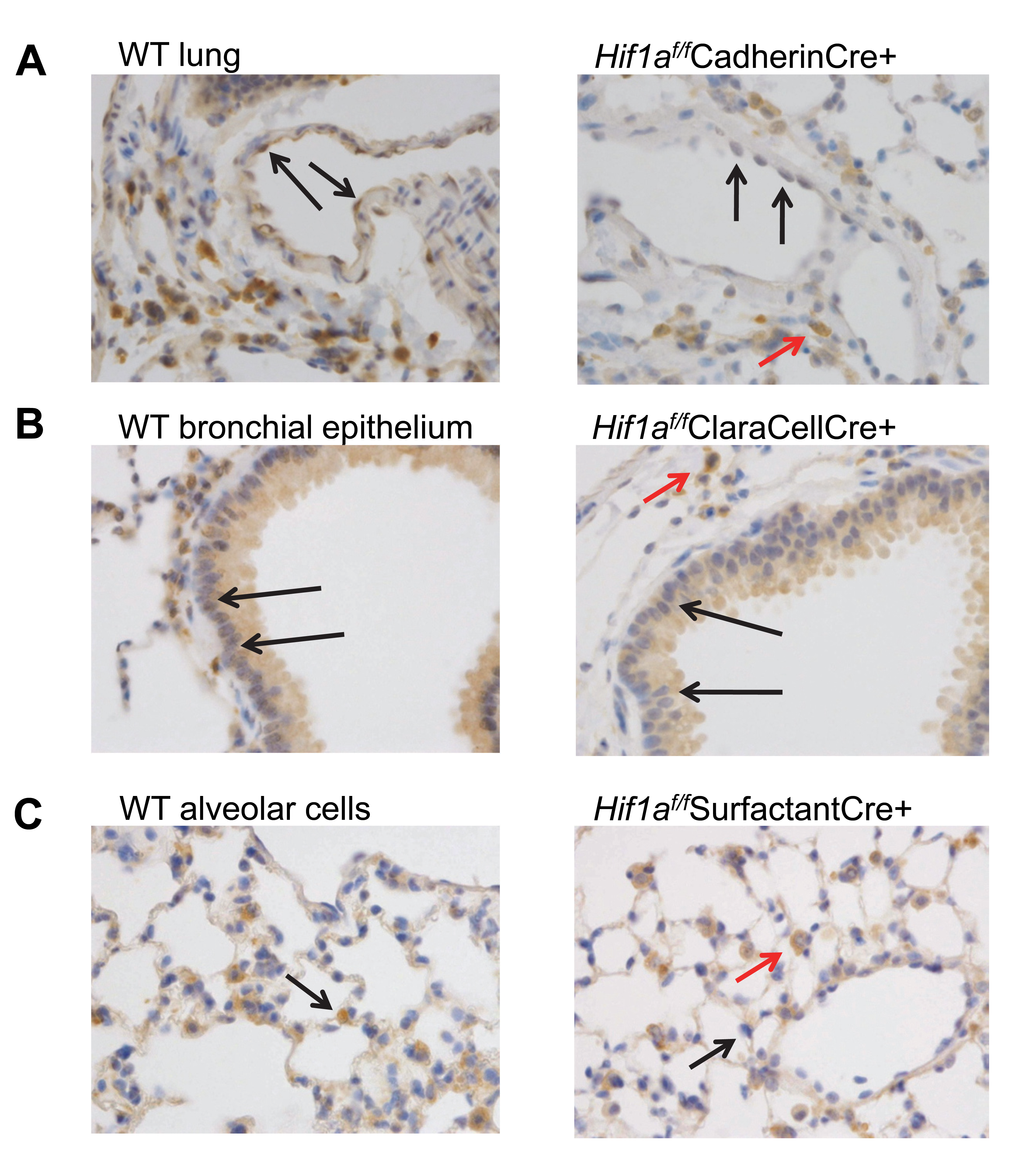

Supplement: Figure S5 — HIF1A immunohistochemistry. Hif1a immunohistochemistry is shown for untreated wild-type (WT, left side) compared to cell-type-specific Hif1a knock-out tissue (right side) including (A) lung endothelium, (B) bronchial epithelium, and (C) alveolar cells. (A) Untreated WT lung endothelium (left) display several nuclei with considerable HIF1a immunopositivity (black arrows). Even after DMOG treatment, lung endothelial cells (black arrows) of Hif1af/fCadherinCre+ animals (right) showed no Hif1a expression confirming the good quality of the knock-out. (B and C) Similar findings were obtained for bronchial epithelia (B, black arrows) and alveolar cells (C, black arrows) showing moderate Hif1a expression in WT animals (left) but absence in corresponding cell-type-specific Hif1a knock-out mice after DMOG treatment. (A–C) Inflammatory cells in cell-type-specific Hif1a knock-out animals, infiltrating inflammatory cells (red arrows) served as internal positive control still exhibiting Hif1a expression. (Original magnification 40× for all images.) (TIFF) [file pbio.1001665.s005.tiff]

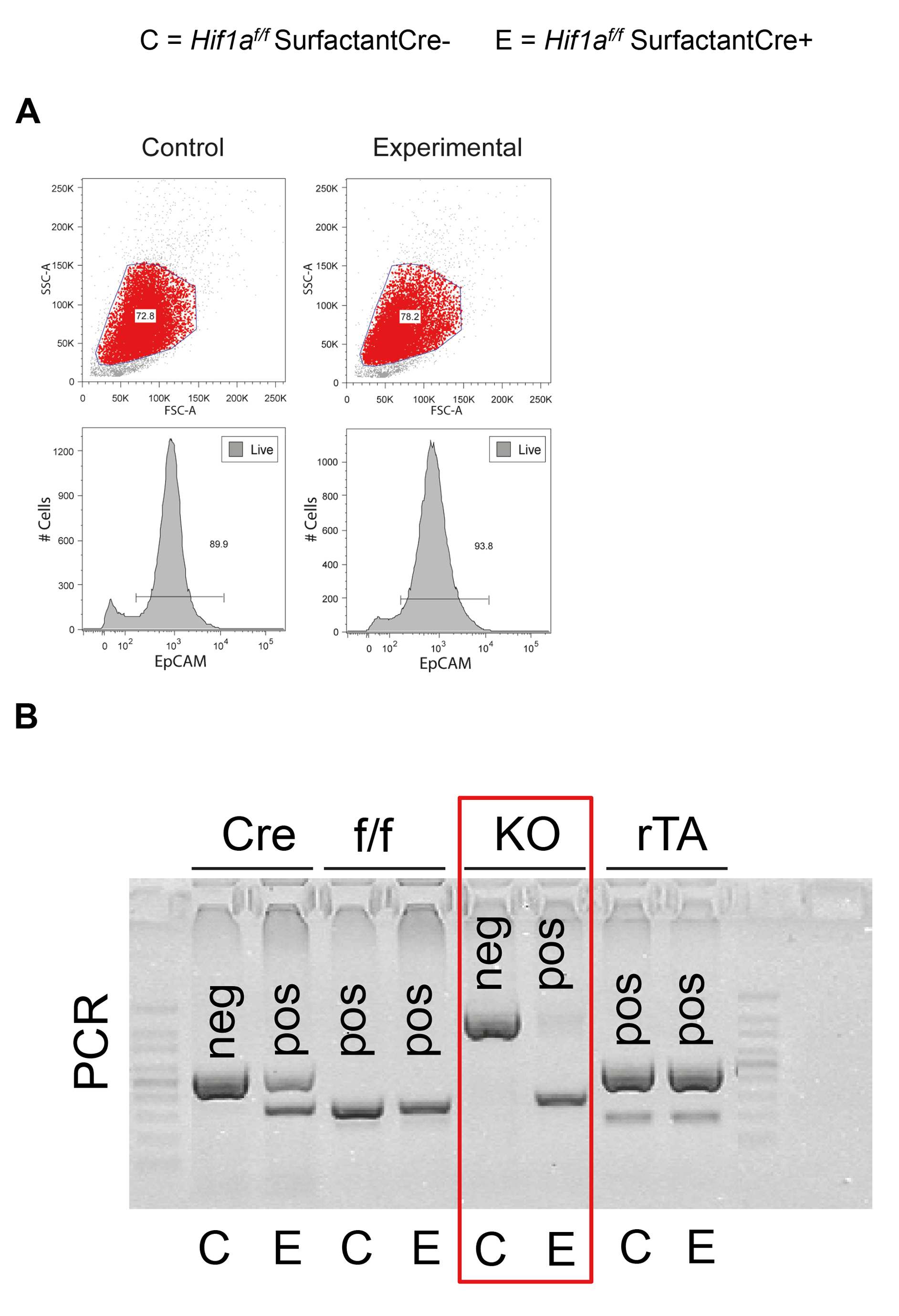

Supplement: Figure S6 — Purified alveolar epithelial cells show reduced HIF1A expression in conditional knockout following VILI. Conditional knockout mice (experimental/E) have a loxP-flanked Hif1a, a SPC-rtTA, and TetO-Cre-recombinase transgene. Littermate controls (control/C) lack the tetO-Cre transgene. (A) Purification of alveolar epithelia cells (AECs) from control and experimental animals: cells are highly enriched for expression of EpCAM (epithelial cell adhesion molecule) indicating purity of AECs. (B) Purified AECs show Cre-recombinase activity and absence of floxed Hif1a gene (red box) in experimental animals determined by conventional PCR using standard genotyping protocols (Jackson Laboratory, by GeneTyper–Mouse Genotyping Service). (TIFF) [file pbio.1001665.s006.tiff]

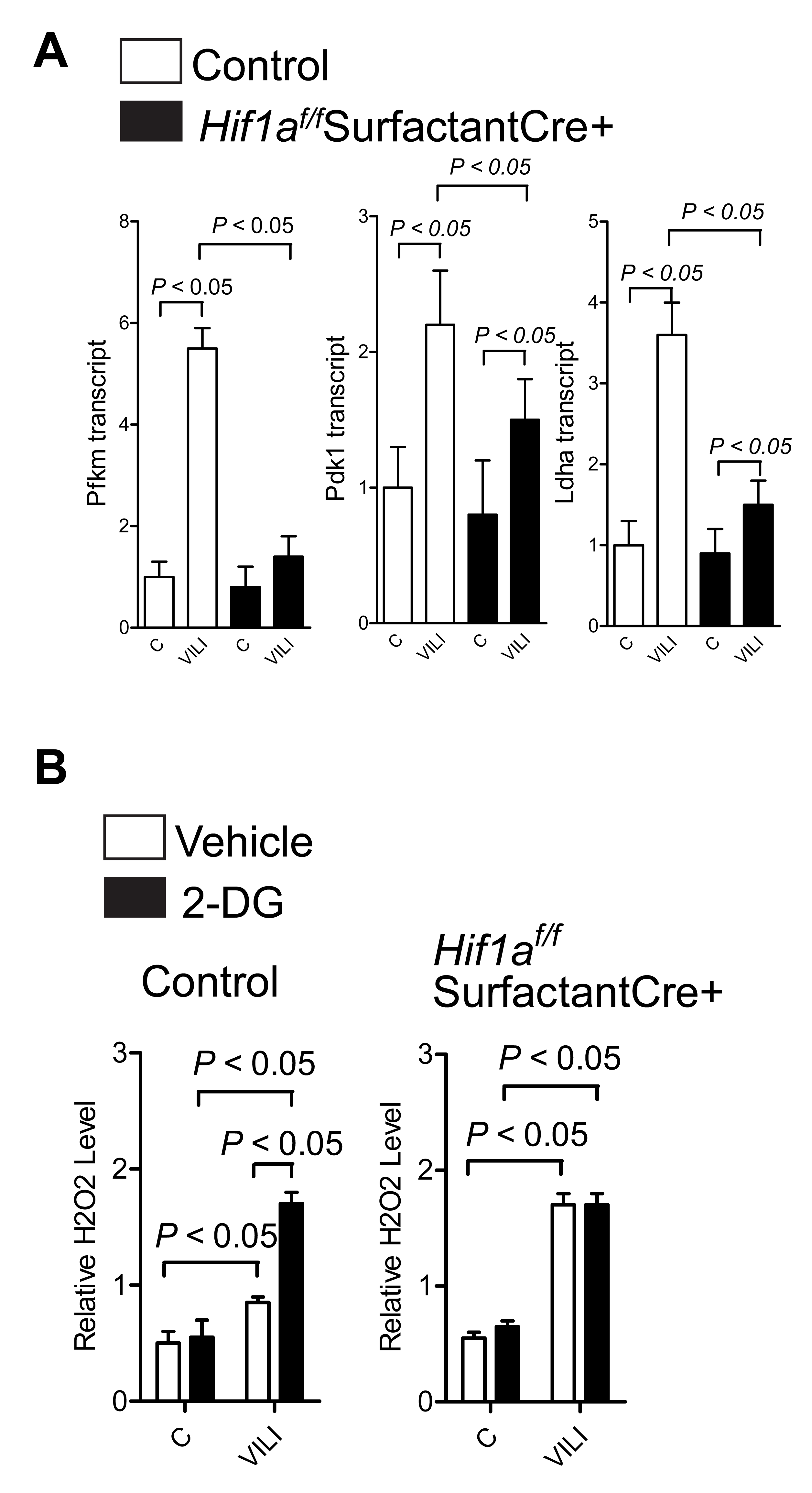

Supplement: Figure S7 — Consequences of glycolysis inhibition during ALI. (A) Transcript levels of phosphofructokinase-m (Pfkm), pyruvate dehydrogenase kinase 1 (Pdk1), and lactate dehydrogenase a (Ldha) from controls (SurfactantCre+) or Hif1af/f SurfactantCre+ mice after 180 min at 45 mbar mechanical ventilation (mean ± s.d., n = 3). (B) Hydrogen peroxide levels of BAL from Hif1af/f SurfactantCre+ mice or age-, gender-, and weight-matched littermate controls (SurfactantCre+) with or without 2-DG treatment. The data are expressed as the mean fluorescence levels from two independent experiments normalized by protein concentration (ELISA). Results are presented as mean ± s.d. (n = 4, unless stated otherwise). (TIFF) [file pbio.1001665.s007.tiff]

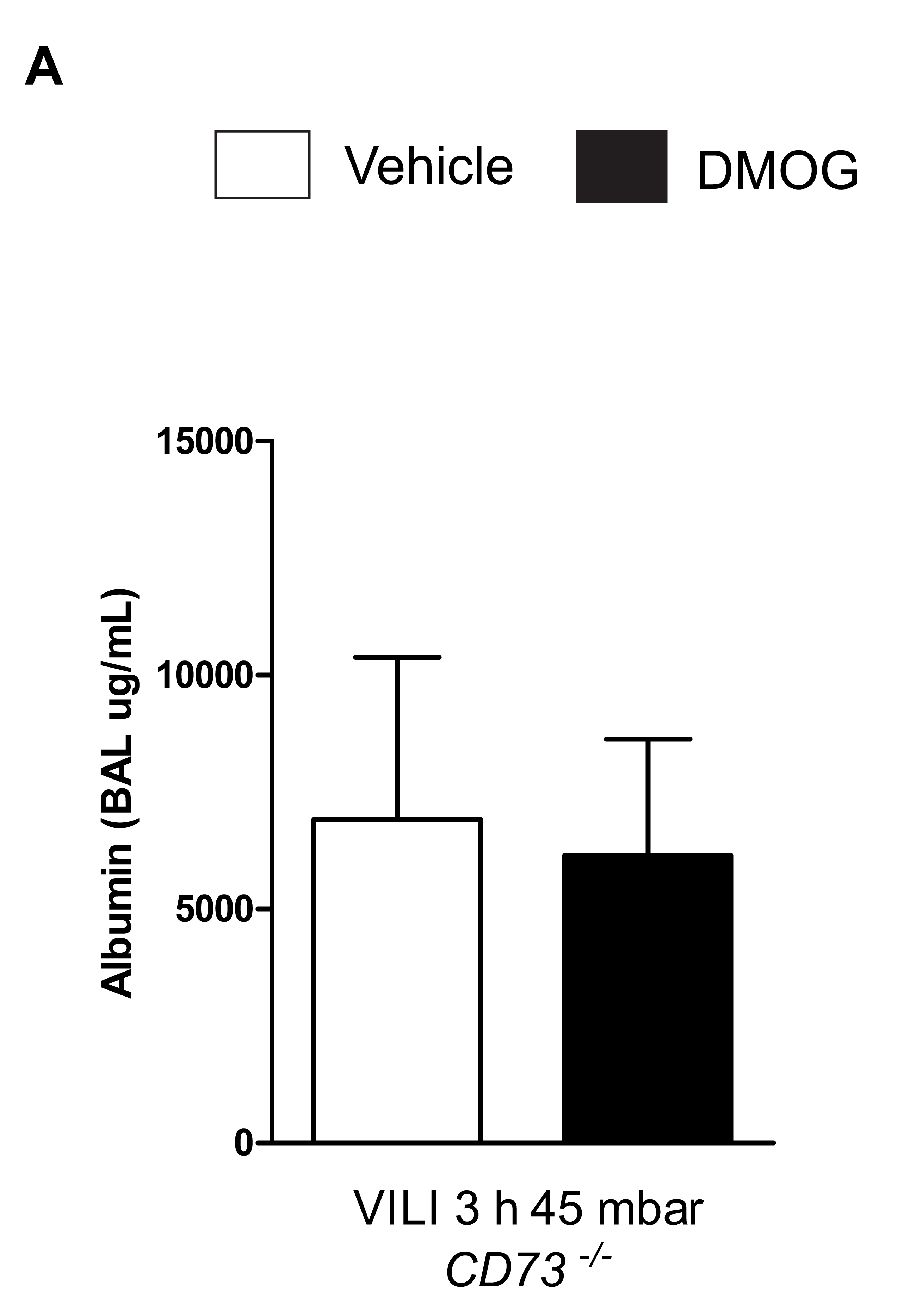

Supplement: Figure S8 — Functional consequences of HIF1A activation during ALI in CD73 deficient mice. (A) HIF1A activator dimethyl-oxaloylglycine (DMOG) during ALI: BL6C57 or CD73−/− mice were treated with 1 mg DMOG or vehicle control 4 h prior to the experimental procedure. Mechanical ventilation was instituted and mice were ventilated for 180 min using pressure-controlled settings (inspiratory pressure of 45 mbar, 100% inspired oxygen concentration). Albumin concentration in the bronchoalveolar fluid (BAL) was determined by enzyme-linked immunosorbent assay. Results are presented as mean ± s.d. (n = 6). (TIFF) [file pbio.1001665.s008.tiff]
